# Transcriptome analysis of PDGFRα^+^ cells identifies T-type Ca^2+^ channel CACNA1G as a new pathological marker for PDGFRα^+^ cell hyperplasia

**DOI:** 10.1371/journal.pone.0182265

**Published:** 2017-08-14

**Authors:** Se Eun Ha, Moon Young Lee, Masaaki Kurahashi, Lai Wei, Brian G. Jorgensen, Chanjae Park, Paul J. Park, Doug Redelman, Kent C. Sasse, Laren S. Becker, Kenton M. Sanders, Seungil Ro

**Affiliations:** 1 Department of Physiology and Cell Biology, University of Nevada School of Medicine, Reno, Nevada, United States of America; 2 Department of Physiology, Wonkwang Digestive Disease Research Institute and Institute of Wonkwang Medical Science, School of Medicine, Wonkwang University, Iksan, Chonbuk, Korea; 3 Sasse Surgical Associates, Reno, Nevada, United States of America; 4 Gastroenterology and Hepatology, Stanford University School of Medicine, Stanford, California, United States of America; Lewis Katz School of Medicine at Temple University, UNITED STATES

## Abstract

Platelet-derived growth factor receptor alpha (PDGFRα)^+^ cells are distributed into distinct morphological groups within the serosal, muscular, and submucosal layers as well as the myenteric and deep muscular plexi. PDGFRα^+^ cells directly interact with interstitial cells of Cajal (ICC) and smooth muscle cells (SMC) in gastrointestinal smooth muscle tissue. These three cell types, SMC, ICC, and PDGFRα^+^ cells (SIP cells), form an electrical syncytium, which dynamically regulates gastrointestinal motility. We have previously reported the transcriptomes of SMC and ICC. To complete the SIP cell transcriptome project, we obtained transcriptome data from jejunal and colonic PDGFRα^+^ cells. The PDGFRα^+^ cell transcriptome data were added to the Smooth Muscle Genome Browser that we previously built for the genome-scale gene expression data of ICC and SMC. This browser provides a comprehensive reference for all transcripts expressed in SIP cells. By analyzing the transcriptomes, we have identified a unique set of PDGFRα^+^ cell signature genes, growth factors, transcription factors, epigenetic enzymes/regulators, receptors, protein kinases/phosphatases, and ion channels/transporters. We demonstrated that the low voltage-dependent T-type Ca^2+^ channel *Cacna1g* gene was particularly expressed in PDGFRα^+^ cells in the intestinal serosal layer in mice. Expression of this gene was significantly induced in the hyperplasic PDGFRα^+^ cells of obstructed small intestine in mice. This gene was also over-expressed in colorectal cancer, Crohn’s disease, and diverticulitis in human patients. Taken together, our data suggest that *Cacna1g* exclusively expressed in serosal PDGFRα^+^ cells is a new pathological marker for gastrointestinal diseases.

## Introduction

In the gastrointestinal (GI) tract, enteric motor neurons coordinate contractile behavior to create productive motor patterns although smooth muscles autonomously generate rhythmic contractile activity independent of neuronal input [1, 2]. Autonomous motor activity and neural regulation are achieved through the integrated activities and responses of smooth muscle cells (SMC), interstitial cells of Cajal (ICC), and platelet-derived growth factor receptor alpha (PDGFRα)^+^ cells (PαC). These cells form an electrical syncytium, collectively known as the SIP (SMC, ICC, and PαC) syncytium. Each type of SIP cell contributes unique behaviors and responses to neurotransmitters, and there may be many more unrecognized behaviors of SIP cells. Remodeling of these cells occurs in a variety of pathophysiological conditions, and the loss, or loss-of-function, of SIP cells can contribute to the development of motor dysfunction [1].

PαC were identified in the GI musculature of mice and humans as KIT-negative fibroblast-like cells [3, 4]. PαC express PDGFRA, the marker for the cells, CD34, a common progenitor cell marker, and a Ca^2+^-activated K^+^ channel, SK3 (KCNN3), all of which are not found in ICC. PDGFRA belongs to the same kinase family as KIT, which is specifically expressed in ICC. ICC and PαC are localized in similar anatomical niches in the serosal, myenteric, intramuscular, and submucosal regions of GI muscles [5, 6]. Both types of interstitial cells, ICC and PαC, are also closely associated with enteric neurons and electrically coupled to SMC [5]. However, the functions of ICC and PαC are distinctly different. Myenteric ICC (ICC-MY) serve as pacemaker cells that generate, and actively propagate, electrical slow waves that are the spontaneous electrical events that lead to phasic contractions of smooth muscles [7–9]. ICC also contribute to responses generated in the SIP syncytium by cholinergic and nitrergic neurotransmitters. PαC mediate inhibitory purinergic neurotransmission in GI smooth muscles [10, 11]. In general, due to the coupling of Ca^2+^-activated Cl^-^ channels to Ca^2+^ release events in ICC [12–14] and coupling of SK3 channels to Ca^2+^-release events in PαC [11, 15, 16], stimuli initiating Ca^2+^ release in these cells will have opposite effects on the excitability of the SIP syncytium: Ca^2+^ mobilization in ICC and PαC will exert excitatory and inhibitory effects on the SMC component of the SIP syncytium.

We have developed methods to separate the three types of SIP cells using transgenic mice that ectopically express green fluorescent proteins (GFP). The isolated cells were then used to obtain the transcriptome that was used to characterize gene expression, providing evidence regarding the specific functional roles of each cell type. We have previously catalogued and characterized the transcriptome of SMC from murine small intestine and colon, and used this data to build the Smooth Muscle Cell Genome Browser [17]. We have done the same analysis of the transcriptome from isolated ICC and added this data to the browser [18]. By analyzing the transcriptomes of SMC and ICC, we have been able to identify many new cell markers and regulatory genes that are related to cell-specific functions.

To complete the SIP transcriptome project, we have characterized the transcriptome of isolated PαC from jejunal and colonic smooth muscle within Pdgfra-eGFP mice [19]. The Smooth Muscle Genome Browser was updated to contain the transcriptome data from PαC and this data has been integrated with genomic level bioinformatics data publically available in the UCSC genome browser [20]. The transcriptome browser now offers a reference for the structure, isoforms, and expression levels of all genes expressed within each of the SIP cells.

By comparatively analyzing the transcriptomes of all SIP cell types, we have identified new selective markers for PαC, including a T-type Ca^2+^ channel, *Cacna1g*, which is specifically expressed in PαC. This Ca^2+^ channel is exclusively expressed in serosal PαC, which may be myofibroblasts, and significantly induced in intestinal partial obstruction, colon cancer, Crohn’s diseases, and diverticulitis.

## Materials and methods

### Animal and tissue preparation

*Pdgfra*^*eGFP/+*^ mice [19] were obtained from Jackson Laboratory. *Pdgfra*^*eGFP/+*^ mice were crossbred with *Myh11*^*Cre-ERT2/+*^ [21] and *Rosa26*^*LacZ/LacZ*^ (Jackson Laboratory) to generate *Pdgfra*^*eGFP/+;*^*Myh11*^*Cre-ERT2/+;*^*Rosa26*^*LacZ/+*^. Intestinal partial obstruction surgeries were performed on one month old *Pdgfra*^*eGFP/+*^ mice and one month old *Pdgfra*^*eGFP/+;*^*Myh11*^*Cre-ERT2/+;*^*Rosa26*^*LacZ/+*^ mice after five consecutive days of intraperitoneal tamoxifen injection as previously described [22]. Jejunal and colonic tunica muscularis, from mice that underwent partial obstruction or sham surgery, were used to isolate PαC through flow cytometry. The animal protocol was approved by the Institutional Animal Care and Use Committee at the University of Nevada-Reno Animal Resources.

### Human tissue preparation

Segments of human colon were obtained from patients undergoing colonic resections due to neoplasm formation or diverticulitis at Renown Medical Center (Reno, NV). Paraffin embedded sections and/or frozen tissue samples of human small intestine, obtained from patients with Crohn’s disease and control normal colon, were obtained from Stanford University School of Medicine, Stanford, California. The Human Subjects Research Committees at Renown Regional Medical Center, and the Biomedical Institutional Review Board at University of Nevada, Reno approved the use of human tissues.

### Flow cytometry and fluorescence-activated cell sorting (FACS)

Cells were dispersed from the tunica muscularis of mouse jejunum/colon and GFP^+^ PαC were sorted from dispersed cells using FACS as previously described [23]. Isolated PαC were lysed and pooled from approximately 30 mice (15 males and 15 females) and used to isolate total RNAs as one collective sample. GFP^+^ PαC were also isolated from partial obstruction surgery mice along with sham operation control mice in a similar manner.

### Isolation of total RNAs

Total RNA was isolated from jejunal PαC (JPαC), and colonic PαC (CPαC) using the mirVana miRNA isolation kit (Life Technologies, Carlsbad, CA). Quality of total RNAs was analyzed via NanoDrop 2000 Spectrometer (Thermo Scientific, Waltham, MA) and 2100 Bioanalyzer (Agilent Technologies, Santa Clara, CA).

### Real time PCR

cDNA libraries were constructed through reverse transcription of the total RNA isolated from FACS-purified PαC as previously described.[24] Reverse-transcription polymerase chain reaction (RT-PCR) and quantitative PCR (qPCR) analyses of cDNA were performed as previously described [24]. All primers used for RT-PCR are shown in S11 Table.

### Construction of RNA-seq libraries and next-generation sequencing

Two RNA-seq libraries were generated and sequenced via Illumina HiSeq 2000 (Illumina, San Diego, CA) following the vendor’s instruction at LC Sciences (Houston, TX) as previously described.[17]

### Bioinformatics data analysis

Paired-end sequencing reads were processed and analyzed as previously described [17]. A cutoff of FPKM = 0.025 generated equal false positive and false negative ratios of reliability. The expression levels of transcripts with FPKM values less than 0.025 was considered to be 0.

### Confocal microscopy and immunohistochemical analysis

Jejunal tissue was analyzed by whole mount and cryostat section staining or GFP fluorescence using confocal microscopy as previously described [25, 26]. Primary antibodies against the following antigens were used: anti-CACNA1G-C (rabbit, 1:50, SantaCruz, TX), anti-CACNA1G-N (rabbit, 1:50, Alomone Labs, Jerusalem, Israel and goat, 1:50, SantaCruz, TX), anti-PDGFR-alpha for mice (goat, 1:100, R&D system, MN), anti-PDGFR-alpha for humans (goat, 1:50, R&D system, MN), anti-PDGFR-beta (goat, 1:200, R&D system, MN), and anti-ACTA2 (rabbit, 1:200, Abcam, MA). Images were collected using the Fluoview FV10-ASW 3.1 Viewer software (Olympus, Tokyo, Japan) with an Olympus FV1000 confocal laser scanning microscope. Cryostat sections were also stained with β-galactosidase using LacZ Tissue Staining Kit (InvivoGen, San Diego, CA).

### Western blot

Protein was extracted from jejunal tissue samples of *Pdgfra*^*eGFP/+*^ mice and human GI tissues. Western blotting was performed as previously described [27]. Primary antibodies against the following antigens were used: anti-CACNA1G-C (rabbit, 1:100, SantaCruz, TX), anti-CACNA1G-N (goat, 1:100, SantaCruz, TX), and GAPDH (rabbit, 1:5000, Cell Signaling, MA).

### Availability of supporting data

The PαC transcriptome was added to the Smooth Muscle Genome Browser [17] in the custom track of the UCSC genome database (UCSC Smooth Muscle Genome Browser) [20]. The browser is available at http://med.unr.edu/physio/transcriptome

(It requires Google Chrome and takes approximately a few minutes to upload the large files). The genome browser contains the transcriptome menus on the “Custom Tracks.” Each menu has different display options.

The abbreviated instructions are as follows: 1) To search transcriptional variants of a gene, type in the gene symbol, and click “go.” 2) Under “Custom Tracks,” select the view option (e.g., “full”) for type of sample (e.g., “PαC Jejunum”), and click “refresh.” 3) Select the bioinformatics data of interest (e.g., click on “full” under “RefSeq Genes” in “Genes and Gene Predictions”), and then click “refresh.” 4) Click “configure” to optimize views (change image width and text size).

The RNA-seq data from this study have been also submitted to the NCBI: jejunal PDGFRαC, GSM1388410 and colonic PDGFRαC, GSM1388411.

### Statistical analysis

qPCR data obtained in the present study was compared using the student’s t test in order to determine whether the differences were statistically significant. Measured variables were expressed as mean ± SEM. The differences in mean values between the two animal groups (sham and hypertrophy) were evaluated and considered significantly different when p ≤ 0.05 or p ≤ 0.01.

## Results

### Identification and isolation of PDGFRα^+^ cells

**Fig 1:**
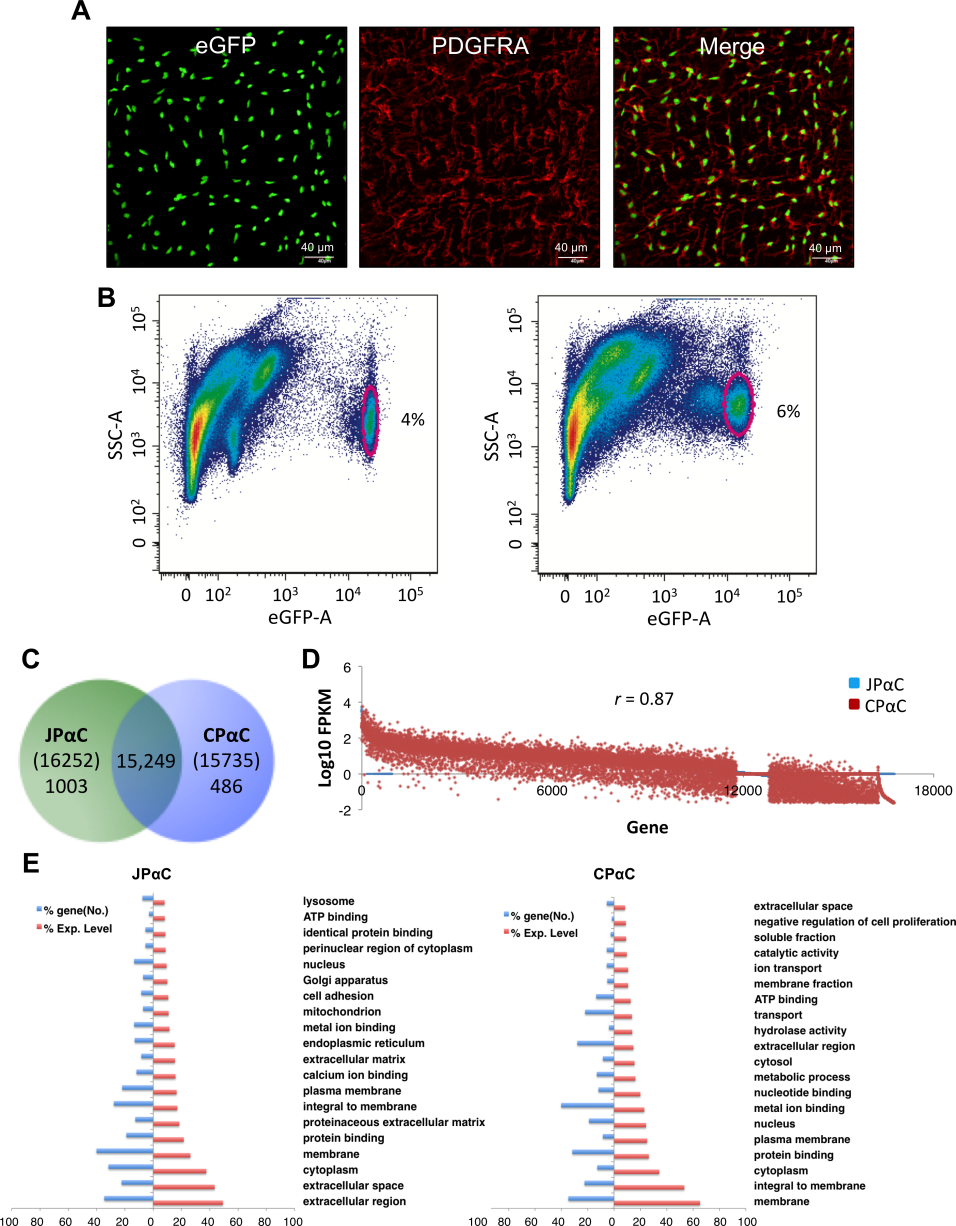
Anaysis of transcriptomes obtained from jejunal and colonic PDGFRα^+^ cells. (A) Identification of PDGFRα^+^ cells in the intestinal smooth muscle with eGFP and a PDGFRA antibody. A z-stack image, obtained through confocal microscopy, of whole-mount jejunum muscularis showing PDGFRα^+^ cells expressing eGFP in the nucleus (*left*). Immunohistochemistry of PDGFRα^+^ cells using a PDGFRA antibody (*middle*). Merged images of eGFP and PDGFRA (*right*). (B) Primary eGFP^+^ PDGFRα^+^ cells from jejunum (*left*) and colon (*right*) identified (circled) by flow cytometry. (C) Venn diagram showing the number of genes identifed in jejunal and colonic PDGFRα^+^ cells (JPαC anc CPαC) by RNA-seq. (D) Comparison of expression levels of genes in JPαC and CPαC. (E) Gene ontologies reported in JPαC and CPαC. The gene ontology (GO: function, process, and component) of PαC-specific genes was analyzed, and key GO terms were compared using normalized expression (FPKM) percentile.

PDGFRα^+^ cells (PαC) were identified by eGFP expression in GI smooth muscle tissues of Pdgfra^eGFP/+^ mice [15, 19] (left panel, Fig 1A). Nuclear eGFP-labeled PαC were confirmed through immunohistochemistry utilizing an anti-PDGFRA antibody (middle panel, Fig 1A). Nuclear eGFP coincided with antibody labeling, as previously demonstrated work [15] (right panel, Fig 1A). Primary PαC from jejunum and colon were further analyzed by flow cytometry. Distinct populations of eGFP^+^ PαC were identified in smooth muscles from the jejunum and colon (Fig 1B). There were at least two distinct groups of jejunal and colonic PαC, which expressed eGFP at relatively high or low levels. eGFP^+^ PαC from jejunal and colonic smooth muscle were only 4–6% of the total events observed by flow cytometry. Therefore, we sorted PαC from 30 mice on the basis of eGFP by FACS, pooled them according to tissue of origin, and isolated mRNAs from each of the two groups of cells.

### Comparison and analysis of transcriptomes in PDGFRα^+^ cells

To identify all genes expressed in PαC in the jejunum and colon, we obtained the PαC transcriptome by performing RNA-seq on the previously mentioned pooled samples. The transcriptome consisted of 16,252 (jejunal PαC) and 15,735 known genes (colonic PαC) (S1 Table). We obtained 137–149 million reads of which 90–91% were mapped to the genome. By gene annotation, we found 48,974 and 48,894 unique gene isoforms in jejunal and colonic PαC, respectively. Complete lists of all the isoforms identified in this study are shown along with tracking ID, gene ID/name, chromosome location, isoform length, and expression levels in both jejunal and colonic PαC (S2 Table). PαC expressed an average of 3 isoforms per gene, produced from different transcription start sites, and/or subject to alternative splicing (S1 Table). Most genes (15,249) were expressed in both jejunal and colonic PαC, but a few hundred genes within each cell-type were specific to the tissue of origin. (Fig 1C). More cell-specific genes were resolved in jejunal PαC (1,003) than in colonic PαC (486). A complete list of the genes expressed in jejunal and colonic PαC are shown with their respective, combined expression levels of all splice variants and numbers of splice variants can be found in S3 Table. Expression levels of all genes in the two PαC groups were compared to each other. Several hundred genes were expressed at high levels (>100 of FKKM) in both PαC groups while genes expressed at low levels (<10 of FKKM) showed a more divergent expression pattern (Fig 1D). Overall expression profiles were similar in PαC from jejunum and colon (correlation coefficient = 0.87). To validate the identity of the cells, markers specific for each cell type (*Pdgfra* for PαC, *Kit* for ICC, and *Myh11* for SMC) were examined. Jejunal and colonic PαC dominantly expressed *Pdgfra* over ICC and SMC (S1A & S1B Fig). Conversely, *Kit* expression was minimal in jejunal and colonic PαC (S1C & S1D Fig). However, jejunal PαC, but not colonic PαC, expressed *Myh11*, but levels were lower than levels shown in SMC (S1E & S1F Fig), suggesting that jejunal PαC may include PDGFRα^+^ SMC precursors [28]. The expression levels of the cell-specific markers validate the authenticity of the RNA-seq data obtained from jejunal and colonic PαC.

To further investigate cell identity and function from our transcriptome data, we analyzed gene ontology (GO) terms of cell specific genes abundantly expressed in each cell type. This analysis revealed key GO terms that distinguish PαC from SMC [17] and ICC [18] (Fig 1E). Key GO terms obtained from the two types of PαC were similar, suggesting they play similar functions in different regions of the GI tract. The most common terms related to structural and extracellular matrix function including plasma membrane, as well as the extracellular region, matrix, and space (Fig 1E).

### Addition to UCSC Smooth Muscle Genome Browser

In an effort to have an interactive transcriptomic profile for each cell type found within the SIP cell types, we previously built a smooth muscle genome browser with jejunal and colonic SMC [17] as well as ICC [18] using the UCSC genome browser (UCSC Smooth Muscle Genome Browser) [20]. We have updated the browser with the PαC transcriptome data, which now contains all three cell types of the SIP (SMC, ICC, and PαC) in jejunal and colonic smooth muscle tissue. This transcriptome browser provides not only the genomic structure of each splice variant (promoter region, exons, and introns) for all genes expressed in PαC (S2 and S3 Tables) as well as SMC and ICC, but also allows for analysis of our transcriptome data using the gene expression and regulation data (ENCODE) [29] that other groups have deposited in the genome database.

### Comparison and analysis of ion channels and transporters expressed in PDGFRα^+^ cells

**Fig 2:**
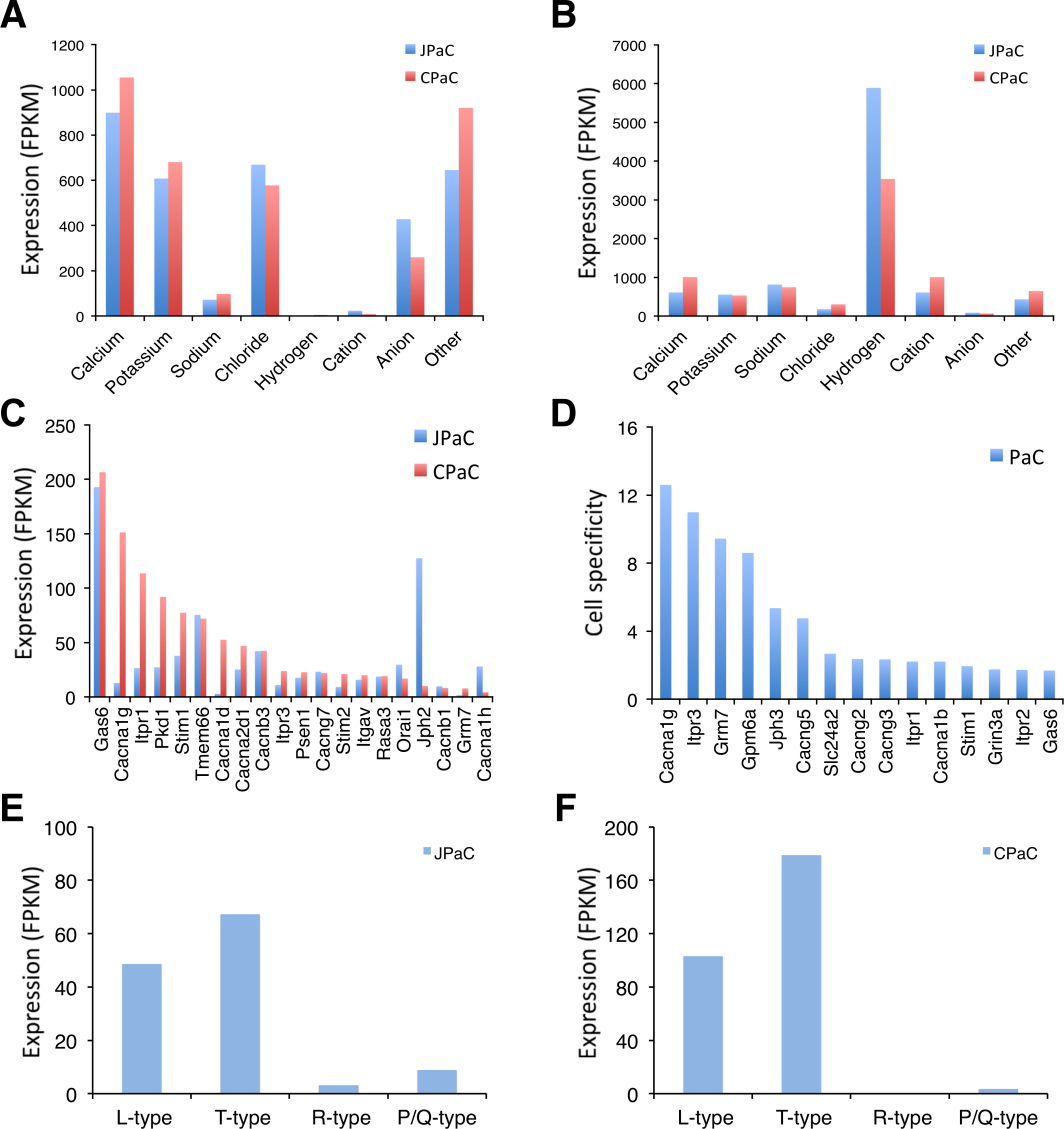
Comparison of ion channel and transporter isoform genes expressed in PDGFRα^+^ cells. (A) Comparison of expression levels of ion channel isoforms in JPαC and CPαC. (B) Comparison of expression levels of ion transproter isoforms in JPαC and CPαC. (C) Ca^2+^ channel isoforms enriched in JPαC and CPαC. (D) PαC-specific Ca^2+^ channel isoforms. Cell specificity was determined by comparative analysis of gene expression profiles among PαC, SMC, and ICC: PαC^expression level (FPKM)^/[SMC^expression level (FPKM)^ + ICC^expression level (FPKM)^]. (E and F) Voltage-dependent Ca^2+^ channel isoforms (L-type: *Cacna1c* & *d*, T-type: *Cacna1h* & *g*, R-type: *Cacna1e*, P/Q-type: *Cacna11*) expressed in JPαC (E) and CPαC (F).

PαC are coupled via gap junctions to SMC, as are ICC to SMC, creating an electrical syncytium (SIP syncytium) that contributes to the regulation of GI motility [1]. Thus, electrophysiological events in one type of SIP cell can affect the excitability of the other cells in the syncytium. From the PαC transcriptome data we identified various ion channels and transporters that are expressed in jejunal and colonic PαC. We identified 513 and 469 ion channel and transporter isoforms expressed in jejunal and colonic PαC, respectively (S4 Table). The most highly expressed type of channels in both PαC is Ca^2+^ channels (Fig 2A). Expression of these channels makes sense in that the major functional conductance observed thus far in PαC is due to small conductance Ca^2+^-activated K^+^ channels encoded by *Kcnn3* (SK3) [6, 15]. Sources of Ca^2+^ are therefore required for activation of SK3. However, to date, no functional voltage-dependent Ca^2+^ currents have been recorded from PαC under the voltage-clamp conditions of the experiments performed on these cells. In addition, hydrogen transporters are the most dominantly expressed transporters in PαCs of jejunum and colon (Fig 2B). The main channel and transporter classes were further analyzed to identify the most abundantly expressed and cell-specific isoforms.

### Identification of a new T-type Ca^2+^ channel *Cacna1g* specifically expressed in PDGFRα^+^ cells

Each type of PαC differentially expresses Ca^2+^ channel isoforms. The cells abundantly express voltage dependent Ca^2+^ channels *Cacna1g* and *Cacna1h* (T-type, Cav3.1 and Cav3.2), *Cacna1d* (L-type, Cav1.2), and Ca^2+^ channel regulators including *Gas6* and *Jph2* (Fig 2C). *Gas6* appears to be the most abundantly expressed in both PαC. However, the L-type and T-type channels are differentially expressed in jejunal and colonic PαC (Fig 2E and 2F). *Cacna1g* and *Cacna1d* are predominantly expressed in colonic PαC while *Cacna1h* is mainly expressed in jejunal PαC (Fig 2C). *Jph2* is also expressed predominantly in jejunal PαC (Fig 2C). Interestingly, *Cacna1g* appears to be the most cell specific to PαC (Fig 2D), thus we selected it for further study.

**Fig 3:**
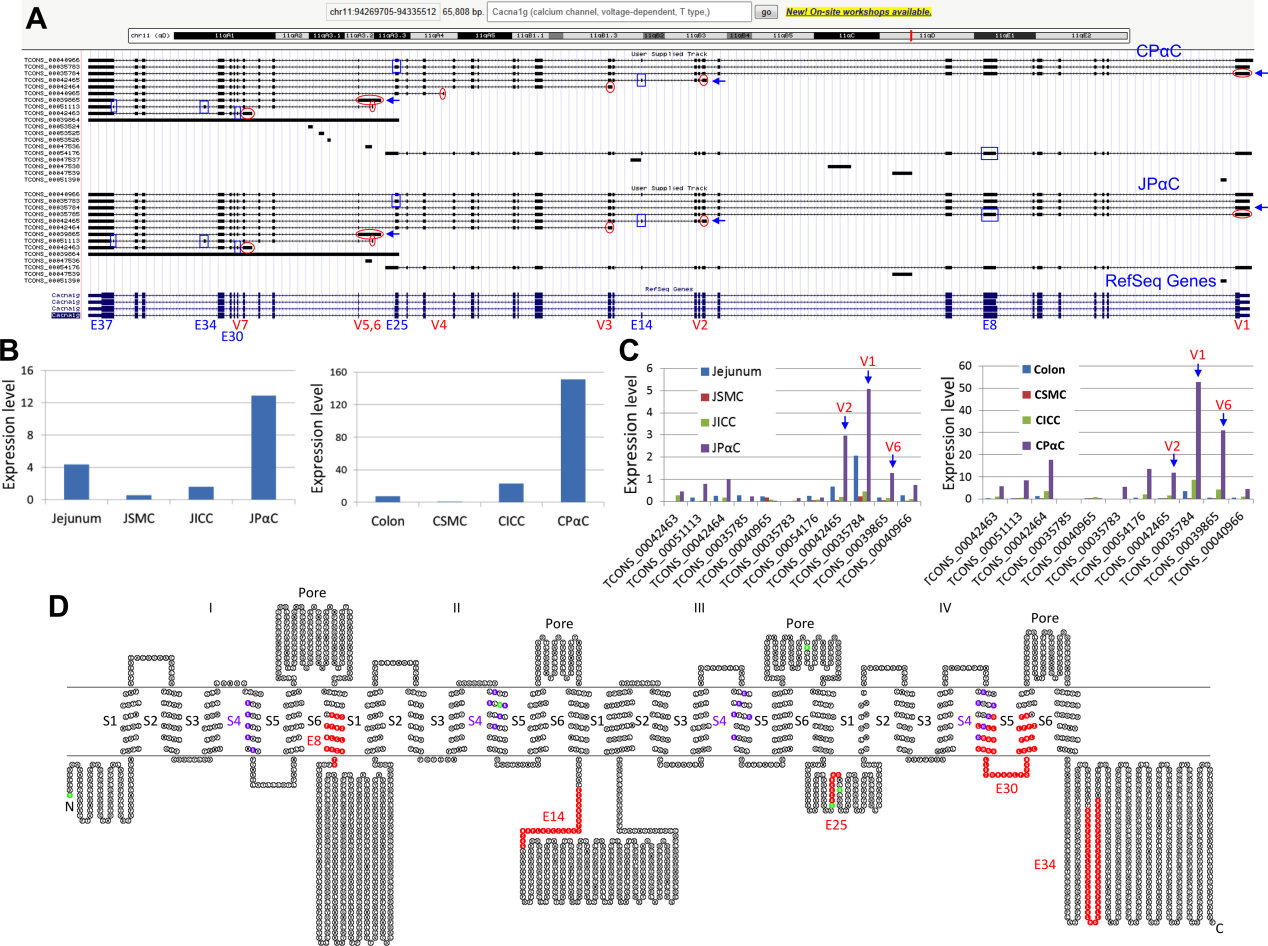
Identification of mutiple *Cacna1g* trancritional variants. (A) A genomic map view of *Cacna1g* variants expressed in JPαC and CPαC. Seven alternative initial exons (V1-7) are circled in red and six differentially spliced exons (E8, E14, E25, E30, E34, and E37) are boxed in blue. (B) Expression (FPKM) levels of total *Cacna1g* mRNAs in JPαC and CPαC. (C) Expression levels of *Cacna1g* transcriptional vaiants in JPαC and CPαC. (D) A topological map of CACNA1G variants. Each circle denotes a single amino acid. Colors on amino acid sequence show distinct regions and domains. Red represents missing, or inserted, peptides from differentially spliced exons. Green represents start codons found in differentially spliced variants. Six transmembrane domains (S1-6) and a pore region are shown.

The *Cacna1g* gene encodes a low voltage-dependent Ca^2+^ channel subunit alpha1 G. The Ca^2+^ channel regulates a variety of Ca^2+^-dependent processes including muscle contraction, secretion, neurotransmission, cell motility, cell division, and cell death [30]. There are 11 transcriptional variants of *Cacna1g* expressed in PαC (Fig 3A and S2 Table). The gene is large and complex, consisting of 37 exons. It contains transcription start sites at seven different exons (V1-7) and alternative splicing occurs at exons 8, 14, 25, 30, 34, and 37 (Fig 3A). *Cacna1g* is expressed in PαC at significantly higher levels than seen in either SMC or ICC from both jejunum and colon (Fig 3B). Major variants expressed in jejunal and colonic PαC appear to be TCONS_00035784 (V1, 8,133 bp), TCONS_00042465 (V2, 5,230 bp), and TCONS_00039865 (V6, 4,201 bp) (Fig 3C and S2 Table). Ten variant cDNAs were retrieved from the browser, an open reading frame for each variant was identified, and all subsequent amino acid sequences were aligned and analyzed (S2 Fig). The channel consists of 4 pore and 24 transmembrane regions (4 S1-S6), each S4 contains 5–6 voltage sensor residues (Fig 3D). The four variant transcripts starting at exon 1 (TCONS_00035784, TCONS_00035783, TCONS_00035785, TCONS_00040966) appear to contain all the pore regions and transmembrane domains, while the other variants are truncated at either the N- or C-terminus (S2 Fig). The four variants contain the differentially spliced exons E8, E14, and E25. Among the four full-length variants, the most dominant variant expressed in both PαC is TCONS_00035784, in which E14 and E25 are missing (Fig 3D).

### PDGFRα^low^ and PDGFRα^high^ cells are proliferative in hypertrophy

**Fig 4:**
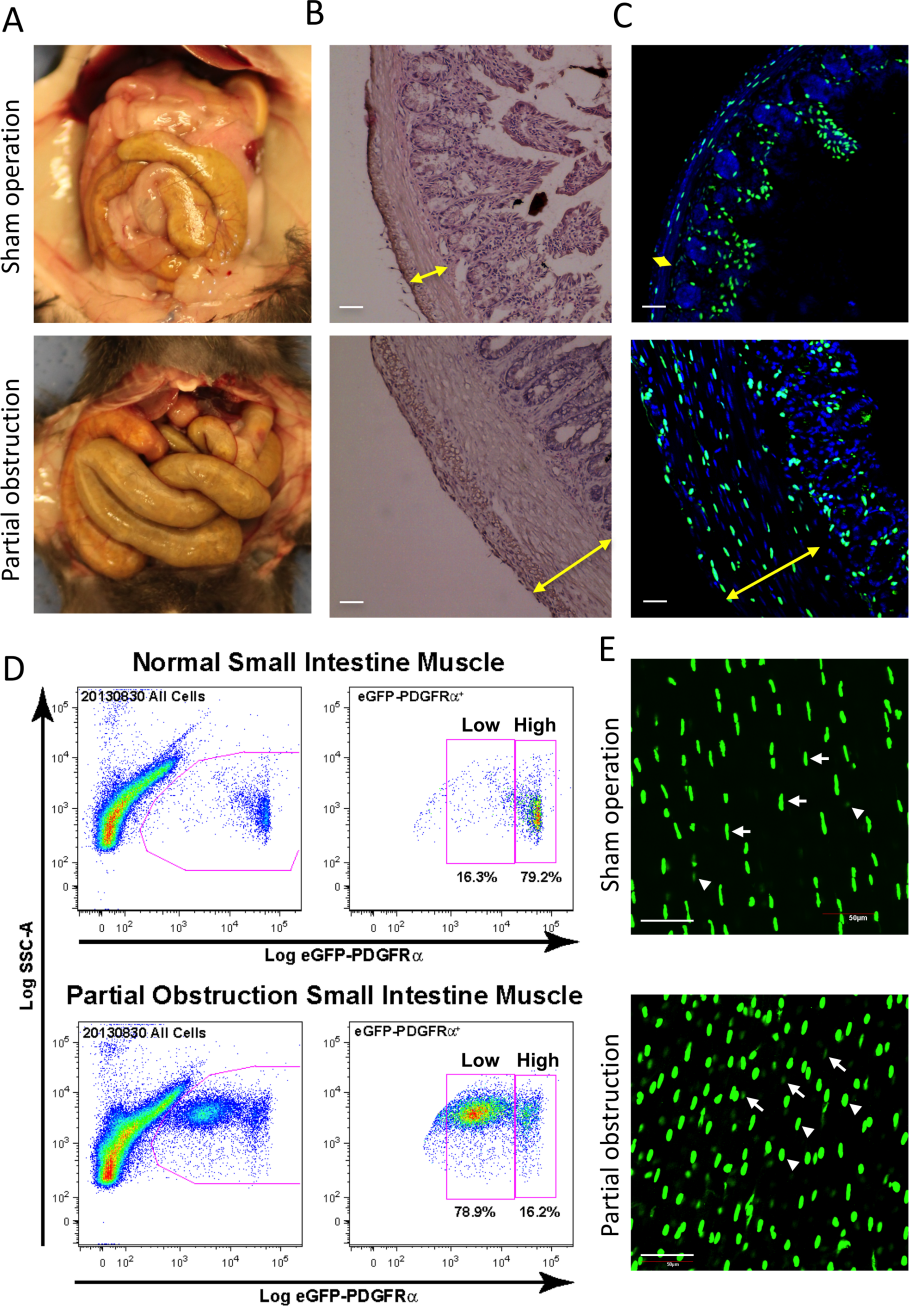
Increased PDGFRα^+^ cells in hypertrophic smooth muscle. Hypertrophic tissue was surgically induced for ~2 weeks by placing a small silicone ring on the distal ileum of transgenic *PDGFRα-eGFP* mice to partially obstruct normal peristaltic movement. (A) Gross images of GI tract in sham and obstruction surgeries. (B) Representative H&E staining of jejunal cross sections from sham control and partially obstructed mice. Hypertrophied jejunum contained significantly thicker circular and longitudinal muscle layers compared to a sham control. Scale bar: 50 μm. (C) Representative confocal laser scanning images of jejunal cross sections from sham operation control and partial obstruction mice showing nuclear eGFP expression in PDGFRα^+^ cells and DAPI (blue) counterstained in the cells. Scale bar: 50 μm. (D) Two populations (eGFP^high^ and eGFP^low^) of primary PDGFRα^+^ cells from hypertrophic jejunum identified by flow cytometry. Note that eGFP^high^ PDGFRα^+^ cells are significantly increased in partial obstruction smooth muscle. (E) A z-stack image, obtained through confocal microscopy, of whole-mount jejunum muscularis from the partial obstruction (bottom) and sham operation control (top) showing eGFP^high^ (arrow heads) and eGFP^low^ (arrows) PDGFRα^+^ cells. Scale bar: 50 μm.

We have previously reported that PαC are highly proliferative during intestinal smooth muscle hypertrophy [22]. Partial obstruction (PO) surgery on the small intestine of mice induced jejunal smooth muscle hypertrophy (Fig 4A). Both circular and longitudinal muscle layers were significantly thicker in PO than in sham operations (SO) (Fig 4B). eGFP^+^ PαC were obviously increased in the muscle layers (Fig 4C). Cytometric analysis identified two distinct populations of cells, eGFP^low^ and eGFP^high^ (Fig 4D). Although both populations were increased in partial obstruction, increases in the number of eGFP^low^ PαC was more dramatic. Increase in the amount of eGFP^low^ and eGFP^high^ PαC in hypertrophic muscle layers was also confirmed in whole mount images (Fig 4E). Since the amount of eGFP correlates with the expression of *Pdgfra*, our data indicates the existence of two populations of PDGFRα^low^ (Pα^low^C) and PDGFRα^high^ cells (Pα^high^C) in hypertrophic smooth muscle where they are highly proliferative.

### All subpopulations of differentially localized PDGFRα^+^ cells are highly proliferative in hypertrophic tissue

**Fig 5:**
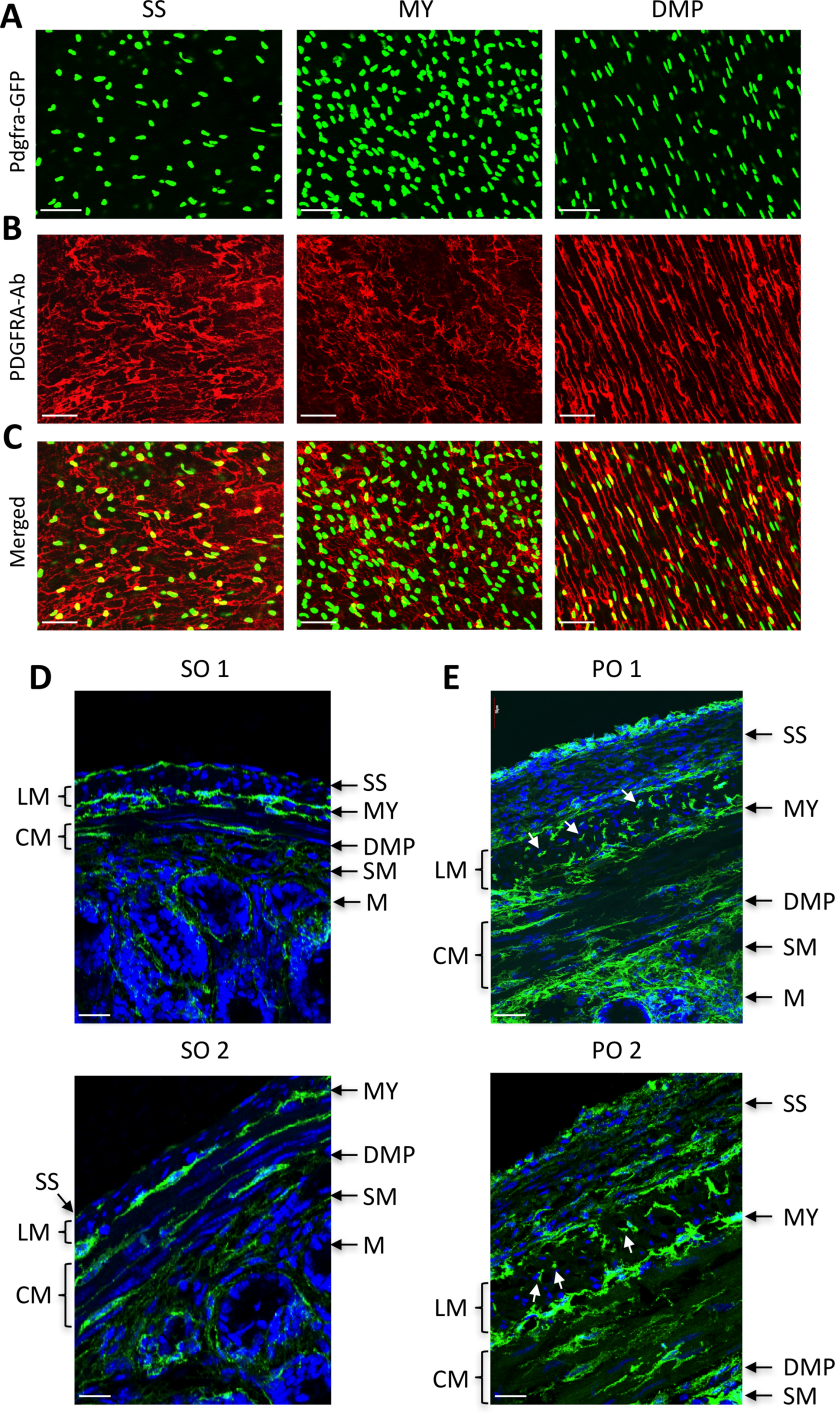
Identification of PDGFRα^+^ cell subpopulations dedifferentiated in hypertrophic smooth muscle. (A-C) Confocal section images of PDGFRα^+^ (Pdgfra-eGFP^+^) cell subpopulations identified in the subserosal layer (SS), myenteric region (MY), and deep muscular plexus (DMP) with PDGFRA antibody (A), eGFP (B) and merged (C) in jejunum. (D and E) Cross section images of PDGFRα^+^ cell subpopulations (green) in sham operation (SO, D) and partial obstruction (PO, E). Proliferating PDGFRα^+^ cells are marked by arrows. LM, lonitudinal muscle; CM, circular muscle; SM, submucosa; M, mucosa. All scale bars are 50 μm.

PαC are differentially localized within the small intestine. Main populations were readily detected in the subserosal (PαC-SS), and myenteric region (PαC-MY), as well as the deep muscular plexus (PαC-DMP) by Pdgfra-eGFP expression levels and immunohistochemical analysis (Fig 5A–5C). The three populations were distinctive in shape and organization. Cross section images revealed three subpopulations localized in distinct regions (Fig 5D). They also showed muscular, submucosal, and mucosal PαC. These subpopulations were well organized in smooth muscle layers in the SO mice, but they were disorganized in hypertrophic tissue induced by PO (Fig 5E). All PαC subpopulations were expanded in the subserosal layer, muscle layers, myenteric region, and deep muscular plexus in PO models. PαC-SS were most expanded, and three distinct subpopulations of cells were detected in the outside layer, middle layer, and inside boundary of the serosal epithelium. Most proliferating PαC were smaller while some cells were hypertrophic. These data suggest that the phenotypes of PαC are dynamic, and they appear to be a major cell type contributing to the remodeling of the tunica muscularis in the hypertrophic response to partial obstruction injury.

### Expression of *Cacna1g* is induced in serosal PDGFRα^+^ cells and dedifferentiated SMC in hypertrophic tissue

**Fig 6:**
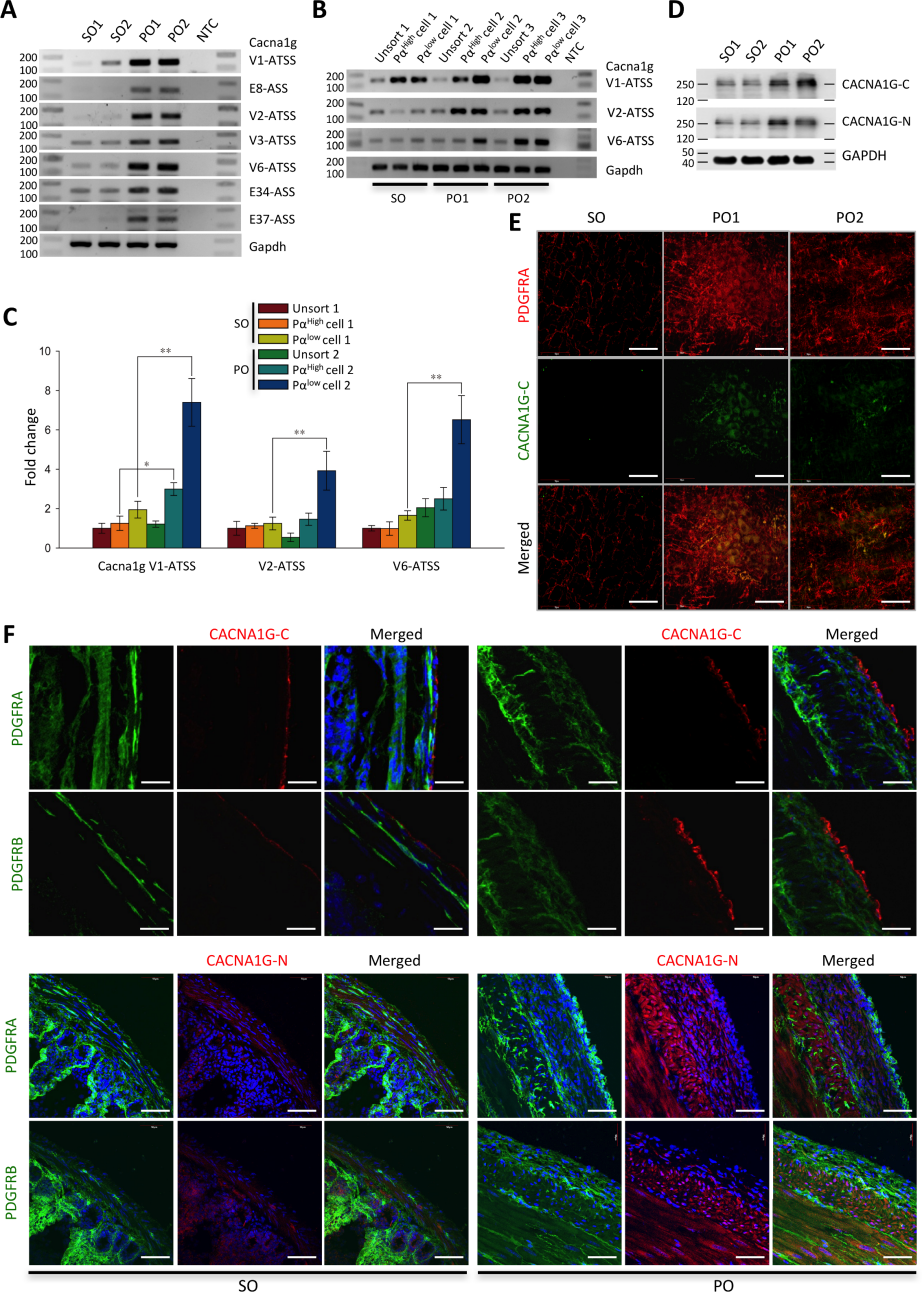
Induced expression of *Cacna1g* mRNAs and protein in hypertrophic smooth muscle. (A) Expression of *Cacna1g* exons with alternative transcriptional start sites (ATSS) and/or alternative spliced sites (ASS) in sham operation (SO) and partial obstruction (PO) examined by RT-PCR. NTC is non-template control. (B) Detection of *Cacna1g* mRNAs in isolated eGFP^high^ and eGFP^low^ PDGFRα^+^ cells from sham operation and partial obstruction opertaion by RT-PCR. PCR products were analyzed with a DNA size marker on 1.5% agarose gels. Note expression of *Cacna1g* is increased in eGFP^high^ and eGFP^low^ PDGFRα^+^ cells. (C) Quantification of *Cacna1g* mRNAs by qPCR. * *p* ≤ *0*.*05* and ** p ≤ *0*.*01*, SO versus PO. (D) Western blot analysis using N-terminal and C-terminal CACNA1G antibodies (CACNA1G-N and CACNA1G-C), showing that the protein has significantly higher expression levels in hypertrophic tissue induced by partial obstruction. (E) Detection of serosal PDGFRα^+^ cells expressing CACNA1G in partial obstruction models by CACNA1G-C antibody. Scale bars are 50 μm (F) Confocal cross section images of serosal PDGFRα^+^ cells in sham operation and partial obstruction screened with CACNA1G-N and CACNA1G-C antibodies co-labeled with PDGFRA and PDGFRB antibodies. Scale bars are 50 μm.

Induced expression of the transcriptional variants of *Cacna1g* in PO-induced hypertrophic tissue was examined by RT-PCR. All the variant exons with four alternative transcriptional start sites and/or three alternative spliced sites were increased in jejunal smooth muscle tissue of PO compared to SO (Fig 6A). Next, we examined the gene induction in isolated Pα^low^C and Pα^high^C by RT-PCR. The expression of the three main variants (see Fig 3C: V1, V2, and V6) was induced in both types of isolated PαC (Fig 6B). Then we measured expression levels of the three variants in the cells by qPCR. mRNA expression levels of all three variants of *Cacna1g* in Pα^low^C and Pα^high^C in PO were significantly higher than in those cells in SO (Fig 6C). Furthermore, the gene was induced at much higher levels in PO Pα^low^C than PO Pα^high^C. Increased expression of the mRNA in PO Pα^low^C also mirrored the CACNA1G protein expression in PO-induced hypertrophic tissue. Both C-terminal (CACNA1G-C) and N-terminal (CACNA1G-N) antibodies detected two bands at ~250 kDa, showing that CACNA1G was expressed at significantly higher levels in PO as compared to SO (Fig 6D). Expression of CACNA1G was examined within the subpopulations of PαC. CACNA1G (detected by CACNA1G-C antibody) appeared to be expressed only in serosal PαC, in which the protein was increased in hypertrophic tissue induced by PO (Fig 6E). Expression and induction in serosal PαC was confirmed through immunohistochemical analysis of cross-sectioned tissue utilizing the CACNA1G-C antibody (Fig 6F). CACNA1G was localized to PαC in the serosal layer. The protein was also co-localized with PDGFRB (PDGFRA partner β subunit) suggesting that CACNA1G is expressed in PDGFRα^+^/β^+^ in the serosal layer. The protein expressing cells were changed in shape and number. In the PO-induced hypertrophic serosal layer, the cells become round and hyperplasic in the outside layer of the enlarged epithelium, as compared to a long spindle shape in SO. Furthermore, we examined localization of the Ca^2+^ channel by CACNA1G-N antibody. However, the N-terminal antibody detected cells in different layers of the muscle. In SO, cells in the serosal, circular muscle, and longitudinal muscle layers were weakly stained by CACNA1G-N antibody (Fig 6F). The N-terminal antibody immunoreactivity was greatly increased in cells within the serosal, circular muscle, and longitudinal muscle layers in PO. CACNA1G-N^+^ cells were colocalized with PDGFRα^+^/β^+^ within the serosal layer (Fig 6F). In addition, CACNA1G-N antibody robustly detected hypertrophic SMC in the circular and longitudinal muscle layers in PO.

**Fig 7:**
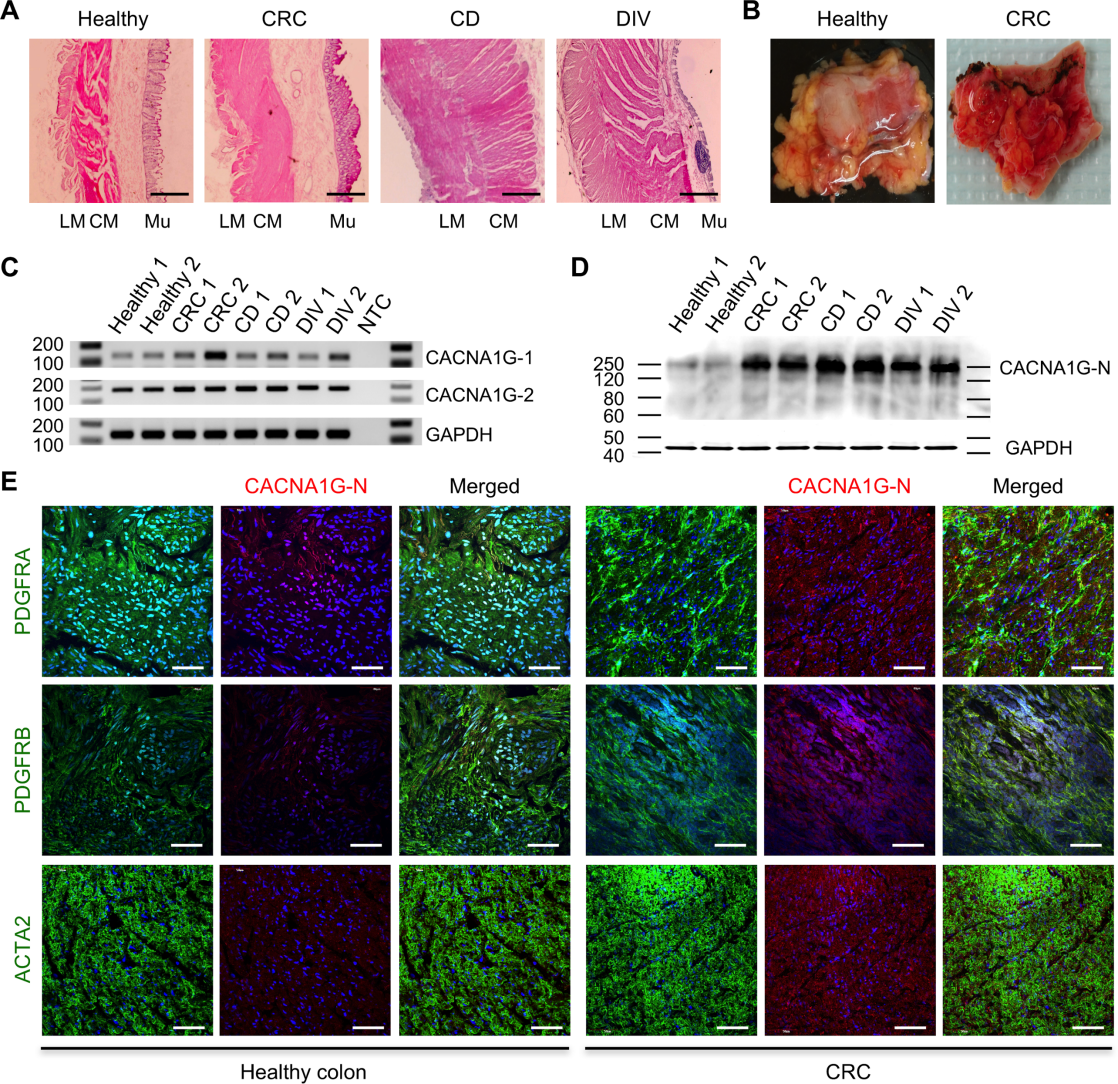
Induced expression of *CACNA1G* in human GI diseases. (A) Anatomical cross-sections of colorectal cancer (CRC), Crohn’s disease (CD) small intestine and diverticulitis (DIV) colon tissue. Marginal colon tissue away from diverticula was used as healthy colon tissue. LM: longitudinal smooth muscle, CM: circular smooth muscle, Mu: mucosa. Scale bars are 100 μm. (B) Inflamed CRC compared to healthy colon tissue. (C) Detection of human *CACNA1G* mRNAs in diseased GI tissues (n = 2). Two independent regions of *CACNA1G* exons (7–8, and 28–29) were amplified by RT-PCR and PCR products were analyzed with a DNA size marker on 1.5% agarose gels. GAPDH gene was used as an endogenous control. (D) Western blot analysis of diseased GI tissues (n = 2) using CACNA1G antibody (N-terminal). (E) Confocal cross section images of PDGFRα^+^ cells screened with CACNA1G antibody (N-terminal) co-labeled with PDGFRA, PDGFRB, and ACTA2 antibodies in CRC and healthy tissues. Scale bars are 50 μm.

The expression levels of *CACNA1G* were also examined in diseased human GI tissues (colorectal cancer, Crohn’s disease small intestine, and diverticulitis colon). The normal colon tissue contained distinct circular and longitudinal smooth muscle layers and healthy mucosa layer while the colon cancer tissue contained hypertrophied muscle layers and cancerous mucosa (Fig 7A). The Crohn’s disease and diverticulitis tissues also showed hypertrophied muscle layers. In addition, the diverticulitis tissue showed a degenerated mucosa layer.

Furthermore, all three disease tissues including colorectal cancer had severe inflammation (Fig 7B). RT-PCR confirmed expression of *CACNA1G* in the human tissues with two primer sets amplifying PCR products spanning the two independent regions (Fig 7C). The CACNA1G protein was detected, with a mass of 250 kDa, and the expression was robustly increased in diseased tissues (Fig 7D). Immunohistochemical analysis (CACNA1G-N) of cross sectioned tissues showed CACNA1G protein localized in PDGFRα^+^/β^+^ cells at low level in healthy colon, but the protein was increased in PDGFRα^+^/β^+^ cells within the hypertrophied smooth muscle of colorectal cancer (Fig 7E). Expression of CACNA1G was also increased in ACTA2^+^ SMC in the hypertrophied smooth muscle. In agreement with the Western blot data, CACNA1G was increased within PDGFRα^+^/β^+^ cells and SMC in the hypertrophied smooth muscle of colorectal cancer.

### Comparative analysis of potassium, cation channel, chloride, and sodium channels

PαC express as many as 92 K^+^ channel subunits (S4 Table). K^+^ inwardly-rectifying channel subfamily J member 8 (*Kcnj8*), intermediate/small conductance Ca^2+^-activated K^+^ channel subfamily N member 3 (*Kcnn3*), ATP-binding cassette sub-family C (CFTR/MRP) member 9 (*Abcc9*), and K^+^ voltage-gated channel subfamily G member 4 (*Kcng4*) were predominantly, but differentially, expressed in JPαC and CPαC. *Kcnn3*, *Abcc9*, and *Kcng4* were more highly expressed in CPαC while *Kcnj8* was more highly expressed in JPαC (S3A Fig). *Kcnn3* was also the most PαC–specific gene (S3B Fig). Cation channel subunits enriched in JPαC and CPαC include aquaporin 1 (*Aqp1*), amine oxidase copper containing 3 (*Aoc3*), and cholinergic receptor, nicotinic beta polypeptide 1 (muscle) (*Chrnb1*). *Aqp1* and *Aoc3* were expressed more in JPαC, but *Chrnb1* was predominantly expressed in CPαC (S3C Fig). PαC–specific cation channel subunits include amiloride binding protein 1 (*Abp1*) and peroxisomal biogenesis factor 5-like (*Pex5l*) (S3D Fig). Both JPαC and CPαC had FXYD domain-containing ion transport regulator 1 (*Fxyd1*) as the most highly expressed cation channel subunit (S3E Fig), as well as Cl^-^ channel Ca^2+^ activated 3 (*Clca3*) and Cl^-^ channel Ca^2+^ activated 2 (*Clca2*) being the most PαC–specific among Cl^-^ channel subunits (S3F Fig). As it pertains to Na^+^ channel expression, Na^+^ channel voltage-gated type VII alpha (*Scn7a*) and Na^+^ channel voltage-gated type I beta (*Scn1b*) were the most predominantly expressed but were differentially expressed between JPαC and CPαC: *Scn7a* was JPαC-dominant and *Scn1b* was CPαC-dominant (S3G Fig). Additionally, *Scn7a* was expressed in the most PαC-specific manner (S3H Fig).

### Comparative analysis of hydrogen transporters

Hydrogen transporter isoforms are the main class of transporters expressed in JPαC and CPαC (Fig 2B). Dominant isoforms expressed in both PαC are H^**+**^ transporting ATP synthase subunits that consist of mitochondrial F1 and F0 complexes (S4 Table). Among them, H^**+**^ ATP synthase mitochondrial F1 complex β (*Atp5b*) and H^**+**^ Transporting, Mitochondrial Fo Complex Subunit F6 (*Atp5j*) were the most abundantly expressed in JPαC and CPαC, respectively (S4A Fig). PαC–specific hydrogen transporter isoforms include ATPase H^**+**^/K^**+**^ transporting non-gastric alpha polypeptide (*Atp12a*) and ATPase H+ transporting lysosomal V0 subunit A4 (*Atp6v0a4*) (S4B Fig).

### Comparative analysis of growth factors, receptors, and transcription factors

PαC expressed 52 growth factors (S5 Table).The dominant growth factors expressed include *Ptn*, *Gpi1*, *Nenf*, *Ogn*, and *Gdf10* which were differentially expressed in JPαC and CPαC (S5A Fig). *Ptn* and *Gdf10* were expressed much higher in CPαC while *Gpi1* and *Ogn* were expressed more in JPαC. However, *Efemp1* and *Ngf* were the most PαC–specific growth factors for both cell types (S5B Fig). PαC expressed as many as 436 receptors (S6 Table). Some receptors that were highly expressed in either JPαC or CPαC were also differentially expressed between the two cell types. *Atp5b* was the most highly and predominantly expressed in JPαC (S5C Fig). Moreover, *App* and *Lgals3bp* were all expressed predominantly in CPαC as compared to JPαC. *Epor* and *Pparg* appeared to be the most PαC–specific receptors in both cell types (S5D Fig). PαC express as many as 134 transcription factors (S7 Table). The most predominantly expressed transcription factors include *Tceb2*, *Ctnnb1*, *Dazap2*, *Jun*, and *Fos* (S5E Fig). Interestingly, *Tceb2*, *Ctnnb1*, and *Dazap2* were expressed higher in JPαC, but *Jun*, and *Fos* were expressed much higher in CPαC (S5E Fig). The most PαC–specific growth factor was *Heyl* (S5F Fig).

### Comparative analysis of epigenetic enzymes and regulators

PαC expressed two DNA methyltransferases (*Dnmt1*, *Dnmt3a*), three *Tet* methylcytosine dioxygenases (*Tet1*, *Tet2*, *Tet3*), aka DNA demethylation enzymes, and a DNA oxidative demethylase (*Alkbh1*) (S8 Table). *Dnmt1* and *Tet2* appeared to be the most predominantly expressed enzymes involved in DNA methylation and demethylation, respectively (S6A Fig). Interestingly all the isoforms were expressed higher in CPαC than in JPαC. *Dnmt1* was also the most cell specific in both cell types (S6B Fig). Furthermore, methyl-CpG-binding domain (MBD) proteins *Mbd3* and *Mbd2* appeared to be the most abundantly expressed MBD among the nine MBD protein isoforms and specifically expressed in both cell types (S6C and S6D Fig). A total of 39 genes expressed in PαC are associated with histone acetyltransferases (S8 Table). Dominant isoforms of histone acetyltransferases include *Taf10*, *Taf9*, and *Ogt* which were differentially expressed in JPαC and CPαC. *Taf10* and *Taf9* were expressed more in JPαC while *Ogt* was expressed at much higher levels in CPαC. (S7A Fig). *Taf10* also appeared to be the most specific to both cell types (S7B Fig). A total of 16 genes with histone deacetyltransferase activity were expressed in PαC (S8 Table). The dominant isoforms of histone deacetyltransferases expressed in PαC include *Hdac5*, *Hdac1*, *Hdac3*, *and Mta2* which were differentially expressed between JPαC and CPαC. *Hdac5* was expressed at a higher level in JPαC while *Hdac1*, *Hdac3*, *and Mta2* were more highly expressed in CPαC (S7C Fig). *Hdac1* also appeared to be the most specific to both cell types (S7D Fig). A total of 24 genes expressed in PαC encode histone methyltransferase isoforms (S8 Table). The most predominantly expressed isoforms of histone methyltransferases include *Setd3*, *Prmt1*, *Ehmt2*, and *Suv420h2* (Kmt5c) which were differentially expressed between JPαC and CPαC. *Setd3* and *Prmt1* were expressed at higher levels in JPαC while *Prmt1 and Suv420h2* were expressed at much higher levels in CPαC (S7E Fig). *Eth1* was the most specific to both cell types (S7F Fig). A total of 15 genes encoding histone demethyltransferases were found to be expressed in PαC (S8 Table). Generally, CPαC expressed higher levels of histone demethyltransferase genes when compared to JPαC. The predominantly expressed isoforms of histone demethyltransferase genes in PαC include *Kdm1b*, *Jmjd6*, *Kdm2a*, and *Phf2* which were differentially expressed between JPαC and CPαC. *Jmjd6*, *Kdm2a*, *and Phf2* had much higher expression in CPαC while *Kdm1b* was expressed at higher levels in JPαC. (S7G Fig). *2410016O06Rik* (*Riox*1, bifunctional lysine-specific demethylase) and *Jmjd6* were the most specific to both cell types (S7H Fig).

### Comparative analysis of protein kinases and phosphatases

PαC expressed 354 protein kinase isoforms (S9 Table) and 105 phosphatase isoforms (S10 Table). The most prominently expressed protein kinases in JPαC include *Mylk* and *Dmpk*, which expressed at much lower levels in CPαC (S8A Fig). The predominantly expressed kinases in CPαC included *Fgfr1* and *Pdgfra* (S9 Table). *Pdgfra* and *Lyn* were the most specific to both cell types (S8B Fig). The most prominently expressed isoforms of phosphatases in PαC include *Ppp1cb*, *Ppp1r12a*, *Ctdsp2*, and *Ppp1cc* and they were also differentially expressed between JPαC and CPαC. *Ppp1cb* and *Ppp1r12a* were expressed more in JPαC while *Ctdsp2* and *Ppp1cc* were expressed much higher in CPαC (S8C Fig). *Ptprn* and Ptprn2 were the most specific to both cell types (S8D Fig).

## Discussion

We have characterized the PαC transcriptome and added this data to our Smooth Muscle Genome Browser that already contains the SMC [17] and ICC transcriptomes [18]. The transcriptomes include all gene isoforms and splice variants expressed in SIP cells (SMC, ICC, and PαC) isolated from the murine jejunum and colon. The transcriptome browser is an interactive database that can be used to search for any gene transcript expressed in SIP cells and can also be used to comparably analyze gene expression and regulation at the level of a single gene using the abundant genome bioinformatics data found at the UCSC genome browser [20]. The browser is open to public and can be accessed at our university website: http://medicine.nevada.edu/physio/transcriptome.

We obtained deep mRNA-seq data (123–238 million reads) from primary SIP cells isolated from the murine jejunum and colon, and identified up to 18,000 genes for each cell type and tissue. The transcriptome accounts for 72% of the total ~25,000 genes encoded in the murine genome. In addition to the massive numbers of genes expressed in SIP cells, each gene is expressed as multiple splice variants (an average of three variants per gene). When the splice variants are considered, the total number of gene isoforms expressed is ~55,000. All variants are generated from alternative start sites and alternative splicing of exons, most of which appear to be cell-specific to each of SIP cells.

A transcriptome acts as a blueprint for gene expression and function. Identification of all gene isoforms and their splice variants in SIP cells is vital to understand the cellular and molecular functions of each gene expressed in these cells. Our transcriptome data revealed multiple isoforms of various gene families that are differentially expressed in each of the three SIP cell types. For genetic studies, dominant isoforms for a gene family should be identified and used. Our transcriptome data will assist to identify candidate isoforms (subunits) of protein complexes relevant to SIP cells, such as ion channels. Furthermore, our data showed that the vast majority of genes are transcribed into multiple splice variants. Most functional studies have been carried out with reference genes and have not considered splice variants. However, our data have shown that multiple splice variants lead to amino acid sequence changes via deletions and insertions of alternative exons (e.g. *Cacna1g* in S2 Fig). The differentially expressed variants in SIP cells may be fundamental to the unique cellular and molecular functions observed in each cell type. Our transcriptome data identify all splice variants, expression levels, and exonic maps in SIP cells which will assist in identifying alternative and dominant variants for each gene for use in future functional and molecular studies.

It is also interesting that all three SIP cells have remarkably similar transcriptomes. SIP cells not only express a vast majority of the same genes (up to 93%), but also show similar expression levels for each gene transcript. These similar gene expression profiles support the idea that the three cell types of SIP share a common developmental lineage. Despite this shared gene expression, there are ~1,500 genes that are specific to each type of SIP cell. These cell-specific genes may separate the SIP cells into their distinctive phenotypic differentiation and functional roles.

By comparatively analyzing the transcriptomes from SIP cells, we have identified new cell markers for each of the SIP cell types. For SMC, we found that *Cnn1*, *Mylk*, *Tpm2*, *Tpm1*, *Des*, and *Myh11* are the most distinctive differentiation markers [17]. SMC are phenotypically dynamic, dedifferentiating into a myofibroblast-like synthetic phenotype in pathological conditions such as hyperplasia and in cell culture conditions [31]. During the transition to a synthetic phenotype, SMC lose expression of contractile proteins. To evaluate SMC phenotype, several individual markers have been reported, but it is still unclear which markers are exclusive for differentiated primary SMC. ACTA2 (α-SMA) and MYH11 have been widely used as markers of SMC differentiation [31]. Premature SMC express ACTA2 while mature SMC express MYH11 [31]. However, our transcriptome data of PαC showed that these SMC marker genes are also expressed in intestinal PαC (S1 Fig). Co-expression of SMC marker genes between SMC and PαC suggests the two cells are in the same lineage of the cell development. Small intestinal SMC are indeed derived from PαC during the embryonic smooth muscle development [28]. Embryonic SMC express both PDGFRA/B and ACTA2 in the circular muscle layer at E13, becoming mature SMC by losing PDGFRA/B at E15 [28], suggesting that PDGFRα^+^/β^+^ cells are SMC precursors. In addition, mature intestinal SMC become PαC when they are dedifferentiated and growing in hypertrophy and culture [22], indicative of phenotypic plasticity between the two cell types. Furthermore, myofibroblasts expressing SMC marker ACTA2, and fibroblast markers including PDGFRA, have been identified in intestinal mucosa subepithelium [23, 32]. Myofibroblasts have both functions of SMC (contractility) and fibroblasts (collagen secretion) [32]. Further studies are needed to phenotypically and functionally distinguish SMC, PαC, and myofibroblasts in GI smooth muscle. Nevertheless, when SMC are phenotypically defined in either pathological or experimental culture conditions, the expression of the three markers PDGFRA, ACTA2, and MYH11 should be evaluated.

For ICC, we have identified the new marker THBS4 [18]. Identification of ICC currently relies on use of KIT antibodies in immunohistochemical analysis. However, KIT expression can be inconsistent for certain ICC phenotypes [33]. For example, ICC lose KIT expression in some GI motility disorders (e.g. diabetic gastroparesis) [34], making it impossible to follow these cells through changing phenotypes via use of KIT antibodies. The new marker, THBS4, may permit the study of ICC long after KIT expression is lost.

Lastly for PαC, we have identified the new cell-specific marker, CACNA1G, a T-type Ca^2+^ channel, in this study. This gene is predominantly expressed in serosal PαC. Interestingly, expression of the protein is increased in proliferative PαC and dedifferentiated SMC in intestinal partial obstruction models. Overexpression of T-type Ca^2+^ channels is associated with various human cancers including colon cancer and esophageal cancer [35,36]. The channels play key roles in proliferation and survival of cancer cells. Silencing the *CACNA1G* gene in colon and esophageal cancer cells inhibits cellular proliferation via a p53-depedent pathway [35,36] indicating that the T-type Ca^2+^ channels induce cancer cell growth. Colorectal cancer originates in the mucosal epithelial cells of the colon [37]. During cancer development, cancer cells grow along with myofibroblasts that drive invasive cancer growth [38]. These tumor-associated myofibroblasts express ACTA2 and PDGFRα/β [39]. We report that proliferative PαC in hypertrophied muscularis from intestinal partial obstruction models and diseased human GI tissue (colorectal cancer tissue, Crohn’s disease small intestine, and diverticulitis colon) overexpress CACNA1G, the same T-type Ca^2+^ channels driving the growth of colon cancer cells, suggesting the Ca^2+^ channels may also induce proliferation of PαC. Moreover, the human GI disease tissues had severe inflammation. Inflammation is known to induce hyperplasia and hypertrophy in intestinal tunica muscularis [40]. Therefore, it is reasonable to speculate that inflammation causes the proliferation of PαC in the muscularis through overexpression of the T-type Ca^2+^ channels, leading to smooth muscle hypertrophy in GI diseases. Since T-type Ca^2+^ channels regulate proliferation, survival and the cell cycle progression of cancer cells, they are good potential targets for anticancer therapy techniques [41], which may be also used for treatment of smooth muscle hypertrophy in the GI tract.

The functional role of the T-type Ca^2+^ channel CACNA1G protein in PαC is mysterious at present. We have not found functional channels or T-like currents in PαC (i.e. no voltage-dependent Ca^2+^ currents are activated in voltage-clamp experiments). PαC are also quite depolarized when isolated, but their membrane potentials may be pulled to more negative potentials when coupled to other cells in the SIP syncytium. However, it is possible that we missed CACNA1G expressing PαC-SS because they are present in lower numbers and display less GFP intensity than the other subtypes, PαC-MY and PαC-DMP, that do not express the channel protein (Figs 5 & 6). In addition, expression of both the channel mRNAs and protein are low in normal or healthy PαC, but increased in hyperplasic PαC in PO models. Thus, the Ca^2+^ channel current should be investigated more in PαC-SS and hyperplasic PαC. Furthermore, the N-terminal and C-terminal antibodies of the channel protein detected different cell populations, suggesting that transcriptional variants are differentially expressed in the cells. Future studies will be needed to see if the transcriptional variants form a functional Ca^2+^ channel and how the protein is trafficking and sub-localized in the cells.

In the intestinal PO condition, PαC become hyperplasic and/or hypertrophic (Fig 5). Hyperplasic PαC are PDGFRα^low^ while hypertrophic PαC are PDGFRα^high^. The intestinal obstruction increased both PDGFRα^low^ and PDGFRα^high^ cells although PDGFRα^low^ cells are more prominent in hypertrophic smooth muscle. Expression of *Cacna1g* is also increased in both PDGFRα^high^ and PDGRα^low^ cells. We have previously reported the SMC are dedifferentiated into proliferative PDGFRα^low^ cells in hypertrophic tissue as the cells lose the SMC master transcription factor SRF [22]. In this study, we found that PαC were increased within the myenteric region and subserosal layer where SMC were not found (Fig 5E), suggesting PαC may be also directly derived from dedifferentiated PαC. However, the cellular origin of increased PDGFRα^low^ and PDGFRα^high^ cells in hypertrophic GI tissue needs further investigation.

One of the functions of PαC is purinergic neurotransmission. Genes related to purinergic signaling, including *P2ry1*, *Kcnn3*, *Adora1*, and *P2rx7*, are abundantly expressed in CPαC [42]. Our SIP cell transcriptome data confirms that the dominant isoforms are *P2ry1* in *P2ry1-14*, *Kcnn3* in *Kcnn1-4*, and *Adora1* in *Adora1-3*, all of which appear to be specifically expressed in both JPαC and CPαC, but minimal in SMC and ICC. However, the dominant isoform of *P2rx1-7* is *P2rx4* in CPαC in the transcriptome. This discrepancy may occur due to the PCR primers amplifying exons that are alternatively spliced or started in the transcriptional variants of the genes. In agreement with this hypothesis, our PαC transcriptome identified four transcriptional variants in *P2rx7* and two transcriptional variants in *P2rx4*. Taken together, the SIPs transcriptome data provides a new tool to search dominant genes involved in various pathways in SIP cells.

Additionally, PαC expressed as many as 52 distinct growth factors (S5 Table). Among them, *Efemp1*, *Ngf*, *Ptn*, and *Gdf10* were expressed in a PαC-specific manner. EFEMP1 (aka, Fibulin-3) contains tandemly repeated epidermal growth factor (EGF)-like repeats. This gene is overexpressed in gastric cancer [43], gliomas [44], ovarian cancer [45], and mesothelioma [46], suggesting PαC may play a role in tumor cell malignancy, invasion and metastasis. Another PαC-specific growth factor, *Ngf*, is required for the survival and maintenance of sympathetic and sensory neurons [47]. PαC are located closely to the terminals of motor neurons in the myenteric and muscular plexuses of the small intestine [48]. This observation suggests PαC may regulate the motor neuronal growth via NGF in the intestine. In addition, *Ngf* is also overexpressed in the majority of solid human tumors, and an anti-cancer therapy that blocks NGF using antibodies is currently being developed [49]. Like *Ngf*, *Ptn* promotes neurite outgrowth in the central nervous system [50]. This gene is likewise overexpressed in many types of cancers including stomach and colon cancer [51]. The last PαC-specific growth factor is *Gdf10* (BMP3b). This growth factor is also involved in neuronal cell development and recovery [52]. Taken together, these accumulated data on the PαC-specific growth factors *Efemp1*, *Ngf*, *Ptn*, and *Gdf10v* suggest PαC play a role in the development of the innervation patterns of myenteric motor neurons and possibly tumorigenesis of some GI cancers.

## Supporting information

S1 Fig Expression of cell marker genes in jejunal and colonic PDGFRα^+^ cells, ICC, and SMC. (A and B) Expression levels (FPKM) of *Pdgfra* (PDGFRα^+^ cells), (C and D) *Kit* (ICC), (E and F) *Myh11* (SMC) in jejunal and colonic PDGFRα^+^ cells, ICC, and SMC.
(TIF)

S2 Fig Alignment of predicted amino acid sequences of CACNA1G transcriptional variants. The open reading frame was identified for each transcriptional variant, and all predicted amino acid sequences were aligned. Six transmembrane helices (S1–S6) in four homologous domains (I-IV) are shown. Colors on amino acid sequence show distinct regions and segments. Green are start codons found in differentially spliced variants. Purple are positively charged residues in S4 voltage sensing segments. Red are missing or inserted peptides from differentially spliced exons.
(DOCX)

S3 Fig Identification of potassium, cation, chloride, and sodium channel subunits highly and specifically expressed in PDGFRα^+^ cells. (A) K^+^ channel isoforms enriched in jejunal and colonic PDGFRα^+^ cells (JPαC and CPαC). (B) PαC-specific K^+^ channel isoforms. (C) Cation channel isoforms enriched in JPαC and CPαC. (D) PαC-specific cation channel isoforms. (E) Cl^-^ channel isoforms enriched in JPαC and CPαC. (F) PαC-specific Cl^-^ channel isoforms. (G) Na^+^ channel isoforms enriched in JPαC and CPαC. (H) PαC-specific Na^+^ channel isoforms. Cell specificity was determined by comparative analysis of gene expression profiles among PαC, SMC, and ICC. Cell specificity was determined by comparative analysis of gene expression profiles among PαC, SMC, and ICC: PαC^expression level (FPKM)^/[SMC^expression level (FPKM)^ + ICC^expression level (FPKM)^].
(TIF)

S4 Fig Identification of hydrogen transporter subunits highly and specifically expressed in PDGFRα^+^ cells. (A) Hydrogen transporter isoforms enriched in JPαC and CPαC. (B) PαC-specific hydrogen transporter isoforms. Cell specificity was determined by comparative analysis of gene expression profiles among PαC, SMC, and ICC.
(TIF)

S5 Fig Identification of growth factors, receptors, and transcription factors highly and specifically expressed in PDGFRα^+^ cells. (A) Growth factor isoforms enriched in JPαC and CPαC. (B) PαC-specific growth factor isoforms. (C) Receptor isoforms enriched in JPαC and CPαC. (D) PαC-specific receptor isoforms. (E) Transcription factor isoforms enriched in JPαC and CPαC. (F) PαC-specific transcription factor isoforms. Cell specificity was determined by comparative analysis of gene expression profiles among among PαC, SMC, and ICC.
(TIF)

S6 Fig Identification of DNA methylation/demethylation enzymes and methyl-CpG binding proteins highly and specifically expressed in PDGFRα^+^ cells. (A) DNA methyltransferases (*Dnmt1* and *Dnmt3a*), methylcytosine dioxygenases (*Tet1*, *Tet2*, *Tet3*), and DNA oxidative demethylase (*Alkbh1*) enriched in JPαC and CPαC. (B) PαC-specific isoforms of DNA methylation and demethylation enzymes. (C) Methyl-CpG binding proteins enriched in JPαC and CPαC. (D) PαC-specific methyl-CpG binding proteins. Cell specificity was determined by comparative analysis of gene expression profiles among PαC, SMC, and ICC.
(TIF)

S7 Fig Identification of histone modifying enzymes highly and specifically expressed in PDGFRα^+^ cells. (A) Histone acetyltransferases enriched in JPαC and CPαC. (B) PαC-specific histone acetyltransferases. (C) Histone deacetylases enriched in JPαC and CPαC. (D) ICC-specific histone deacetylases. (E) Histone methyltransferases enriched in JPαC and CPαC. (F) PαC-specific histone methyltransferases. (G) Histone demethylases enriched in JPαC and CPαC. (H) PαC-specific histone demethylases. Cell specificity was determined by comparative analysis of gene expression profiles among PαC, SMC, and ICC.
(TIF)

S8 Fig Identification of protein kinases and phosphatases highly and specifically expressed in PDGFRα^+^ cells. (A) Protein kinases enriched in JPαC and CPαC. (B) PαC-specific protein kinases. (C) Phosphatases enriched in JPαC and CPαC. (D) PαC-specific phosphatases. Cell specificity was determined by comparative analysis of gene expression profiles among PαC, SMC, and ICC.
(TIF)

S1 Table Summary of transcriptomes obtained from jejunal and colonic PDGFRα+ cells. (XLSX)

S2 Table List of transcriptional variants expressed in jejunal and colonic PDGFRα^+^ cells. (XLSX)

S3 Table List of genes expressed in jejunal and colonic PDGFRα^+^ cells. (XLSX)

S4 Table List of ion channels and transporters expressed in jejunal and colonic PDGFRα^+^ cells. (XLSX)

S5 Table List of growth factors expressed in jejunal and colonic PDGFRα^+^ cells. (XLSX)

S6 Table List of receptors expressed in jejunal and colonic PDGFRα^+^ cells. (XLSX)

S7 Table List of transcription factors expressed in jejunal and colonic PDGFRα^+^ cells. (XLSX)

S8 Table List of epigenetic enzymes and regulators expressed in jejunal and colonic PDGFRα^+^ cells. (XLSX)

S9 Table List of protein kinases expressed in jejunal and colonic PDGFRα^+^ cells. (XLSX)

S10 Table List of phosphatases expressed in jejunal and colonic PDGFRα^+^ cells. (XLSX)

S11 Table Oligonucleotides used in this study. (XLS)

## Abbreviations

GI: gastrointestinal
PαC: platelet-derived growth factor receptor alpha (PDGFRα)^+^ cells
SMC: smooth muscle cells
ICC: interstitial cells of Cajal
SIP cells: SMC, ICC, and PDGFRα^+^ cells
PαC-SS: subserosal PDGFRα^+^ cells
PαC-MY: myenteric region PDGFRα^+^ cells
PαC-DMP: deep muscular plexus PDGFRα^+^ cells
CACNA1G: T-type Ca^2+^ channel alpha 1G subunit
GFP: green fluorescent proteins
GO: gene ontology
RT-PCR: reverse-transcription polymerase chain reaction
qPCR: quantitative PCR
