# Supplementary figures and images for: Transcriptome analysis of PDGFRα+ cells identifies T-type Ca2+ channel CACNA1G as a new pathological marker for PDGFRα+ cell hyperplasia
Source: PLoS One. 2017 Aug 14;12(8):e0182265. doi: 10.1371/journal.pone.0182265 (PMC5555714; doi:10.1371/journal.pone.0182265)

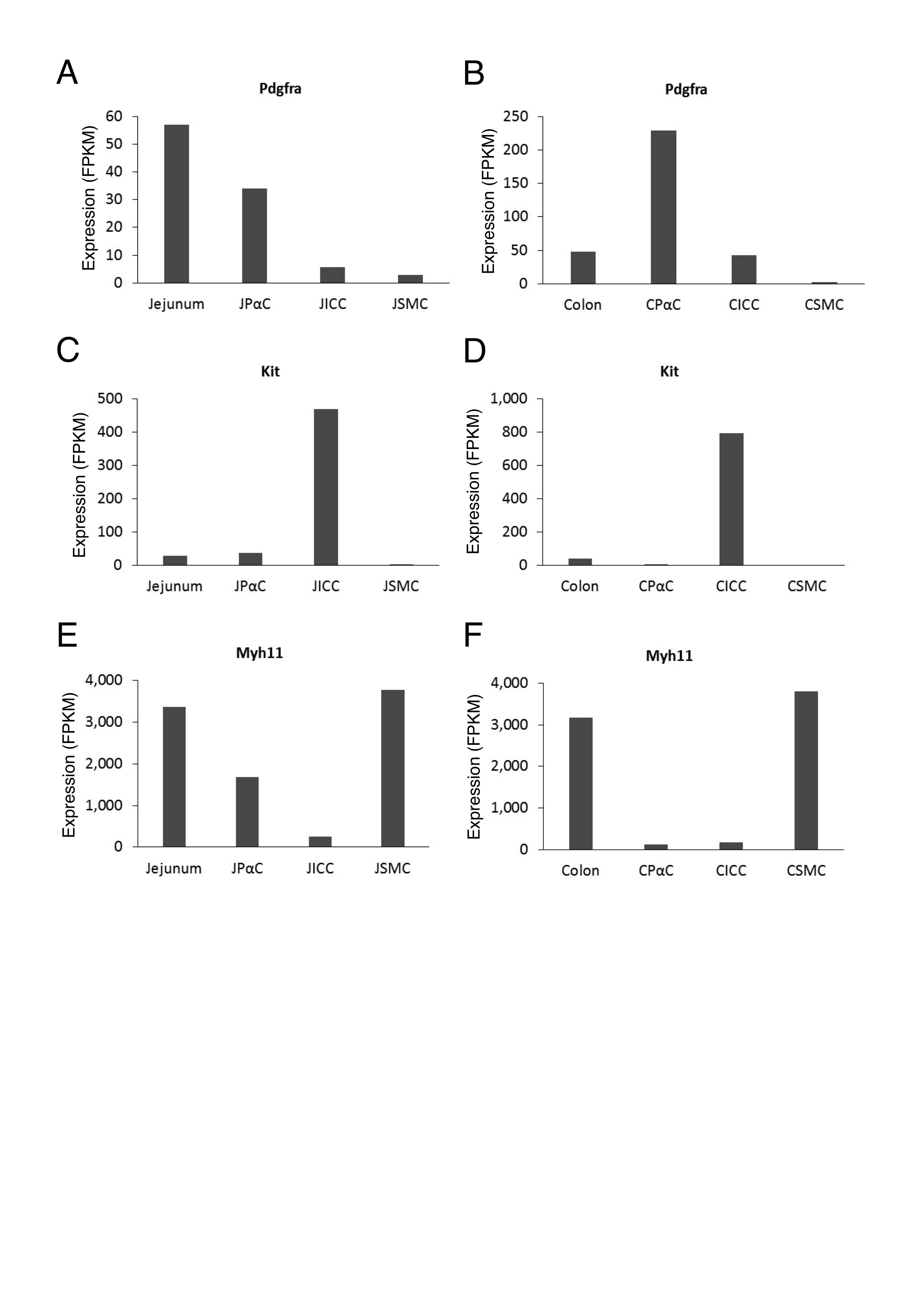

Supplement: S1 Fig — (A and B) Expression levels (FPKM) of Pdgfra (PDGFRα+ cells), (C and D) Kit (ICC), (E and F) Myh11 (SMC) in jejunal and colonic PDGFRα+ cells, ICC, and SMC. (TIF) [file pone.0182265.s001.tif]

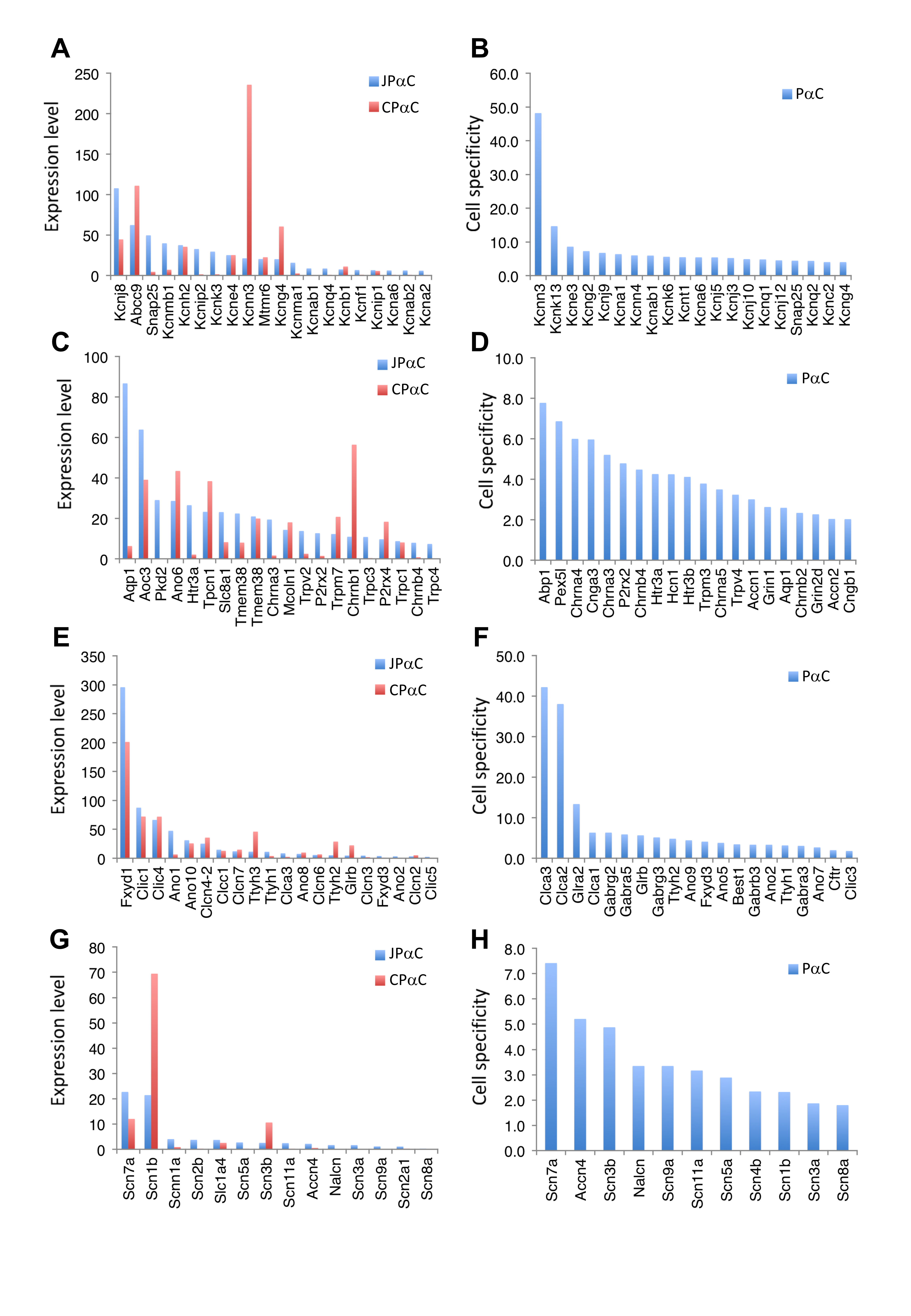

Supplement: S3 Fig — (A) K+ channel isoforms enriched in jejunal and colonic PDGFRα+ cells (JPαC and CPαC). (B) PαC-specific K+ channel isoforms. (C) Cation channel isoforms enriched in JPαC and CPαC. (D) PαC-specific cation channel isoforms. (E) Cl- channel isoforms enriched in JPαC and CPαC. (F) PαC-specific Cl- channel isoforms. (G) Na+ channel isoforms enriched in JPαC and CPαC. (H) PαC-specific Na+ channel isoforms. Cell specificity was determined by comparative analysis of gene expression profiles among PαC, SMC, and ICC. Cell specificity was determined by comparative analysis of gene expression profiles among PαC, SMC, and ICC: PαCexpression level (FPKM)/[SMCexpression level (FPKM) + ICCexpression level (FPKM)]. (TIF) [file pone.0182265.s003.tif]

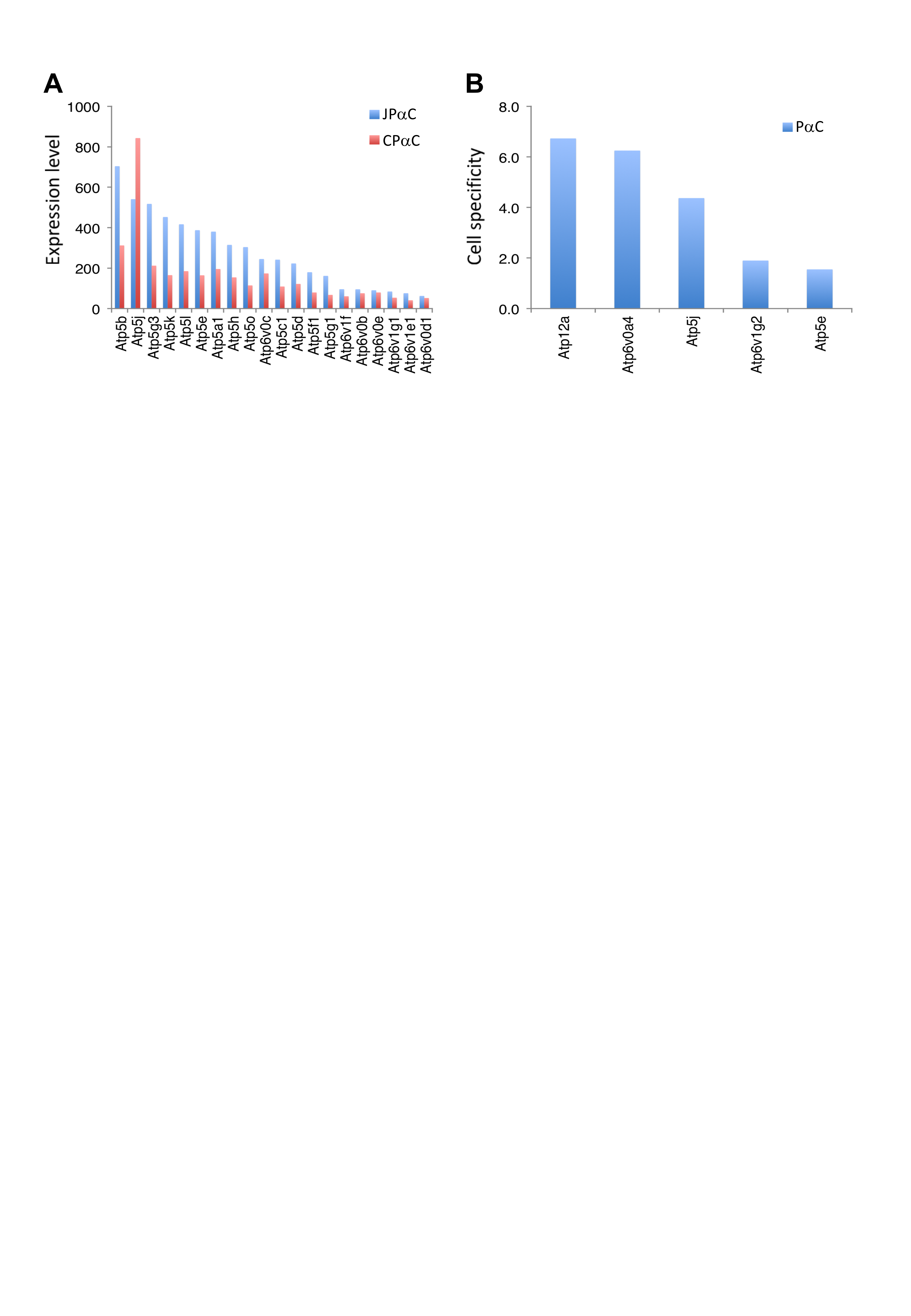

Supplement: S4 Fig — (A) Hydrogen transporter isoforms enriched in JPαC and CPαC. (B) PαC-specific hydrogen transporter isoforms. Cell specificity was determined by comparative analysis of gene expression profiles among PαC, SMC, and ICC. (TIF) [file pone.0182265.s004.tif]

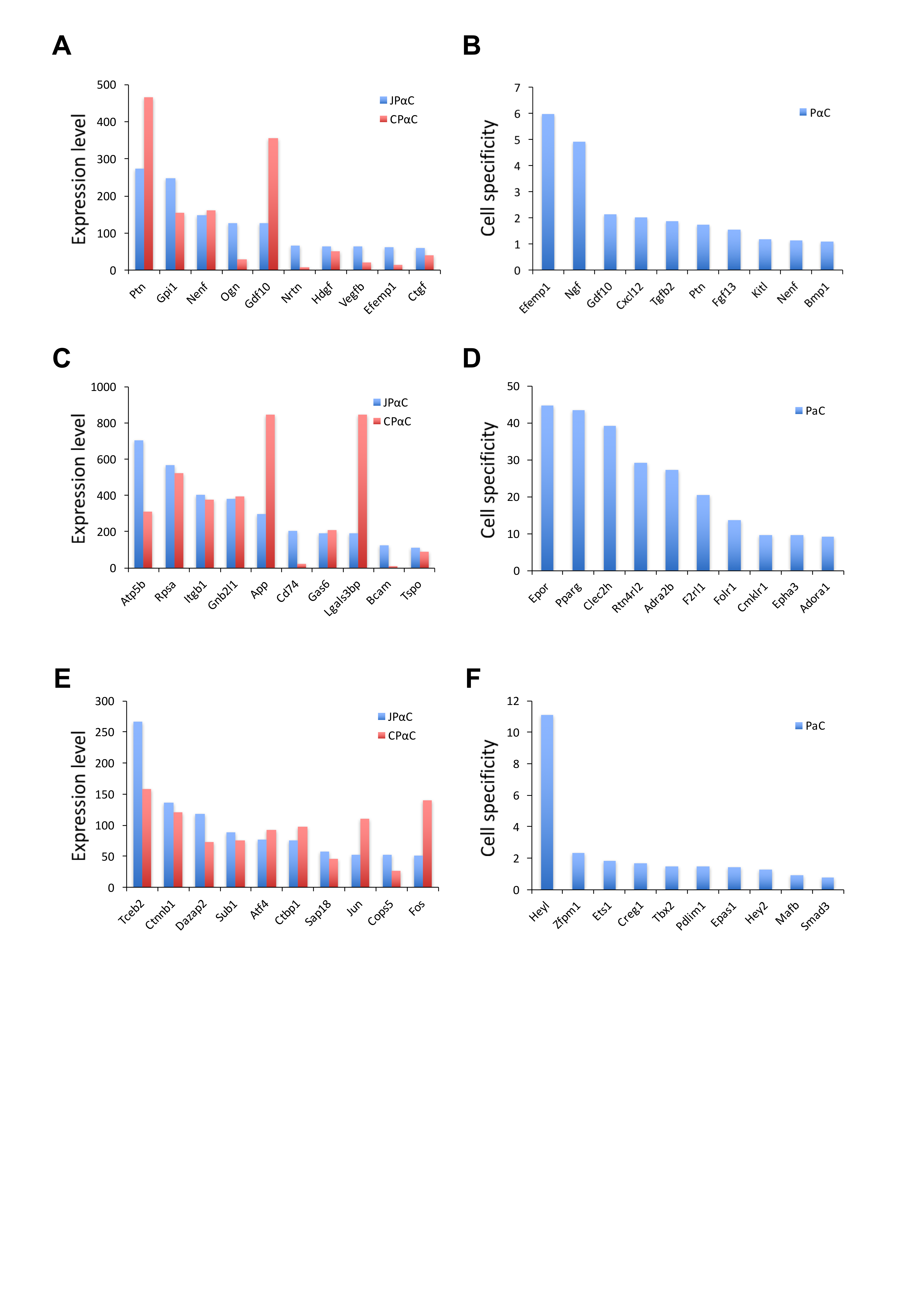

Supplement: S5 Fig — (A) Growth factor isoforms enriched in JPαC and CPαC. (B) PαC-specific growth factor isoforms. (C) Receptor isoforms enriched in JPαC and CPαC. (D) PαC-specific receptor isoforms. (E) Transcription factor isoforms enriched in JPαC and CPαC. (F) PαC-specific transcription factor isoforms. Cell specificity was determined by comparative analysis of gene expression profiles among among PαC, SMC, and ICC. (TIF) [file pone.0182265.s005.tif]

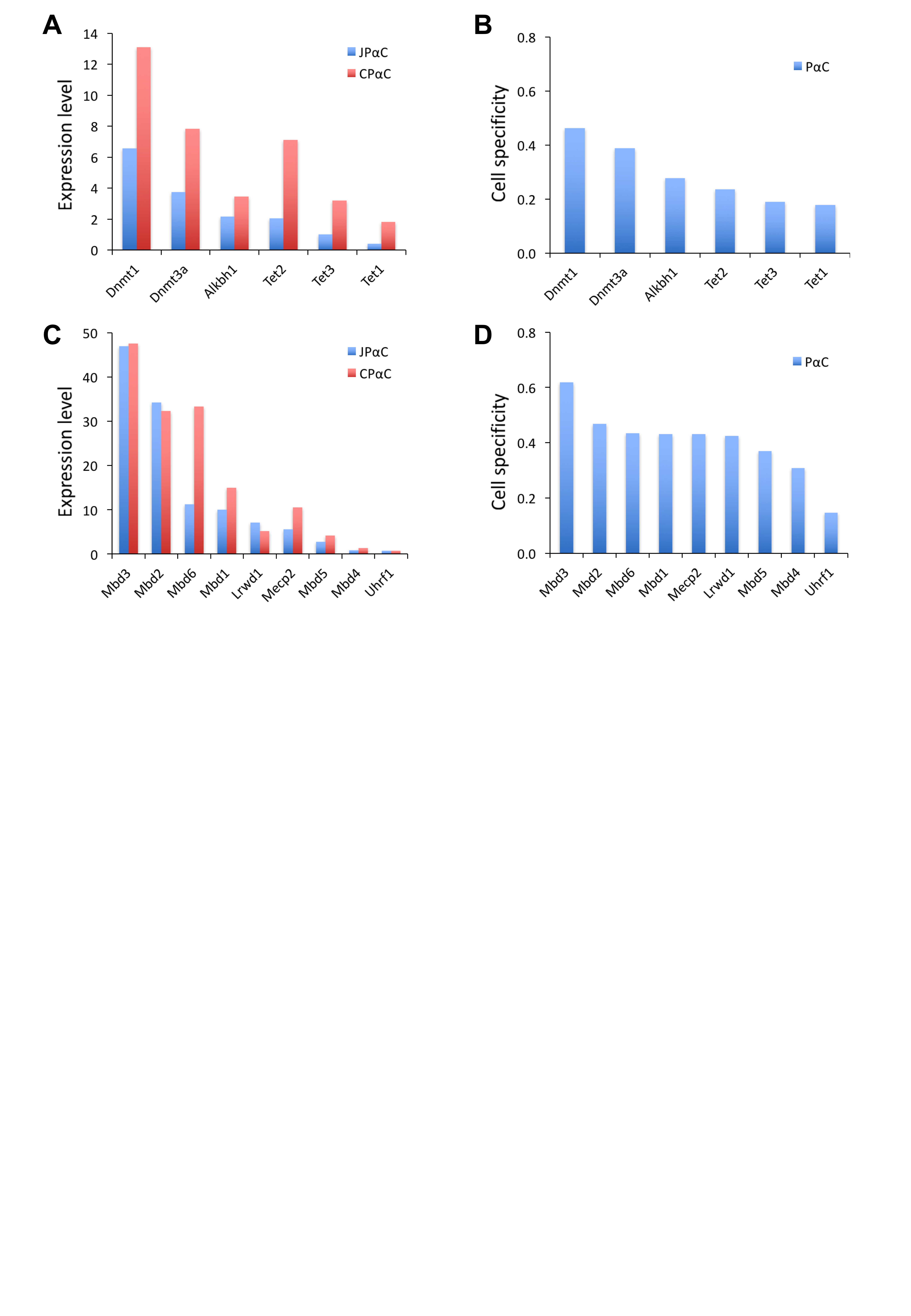

Supplement: S6 Fig — (A) DNA methyltransferases (Dnmt1 and Dnmt3a), methylcytosine dioxygenases (Tet1, Tet2, Tet3), and DNA oxidative demethylase (Alkbh1) enriched in JPαC and CPαC. (B) PαC-specific isoforms of DNA methylation and demethylation enzymes. (C) Methyl-CpG binding proteins enriched in JPαC and CPαC. (D) PαC-specific methyl-CpG binding proteins. Cell specificity was determined by comparative analysis of gene expression profiles among PαC, SMC, and ICC. (TIF) [file pone.0182265.s006.tif]

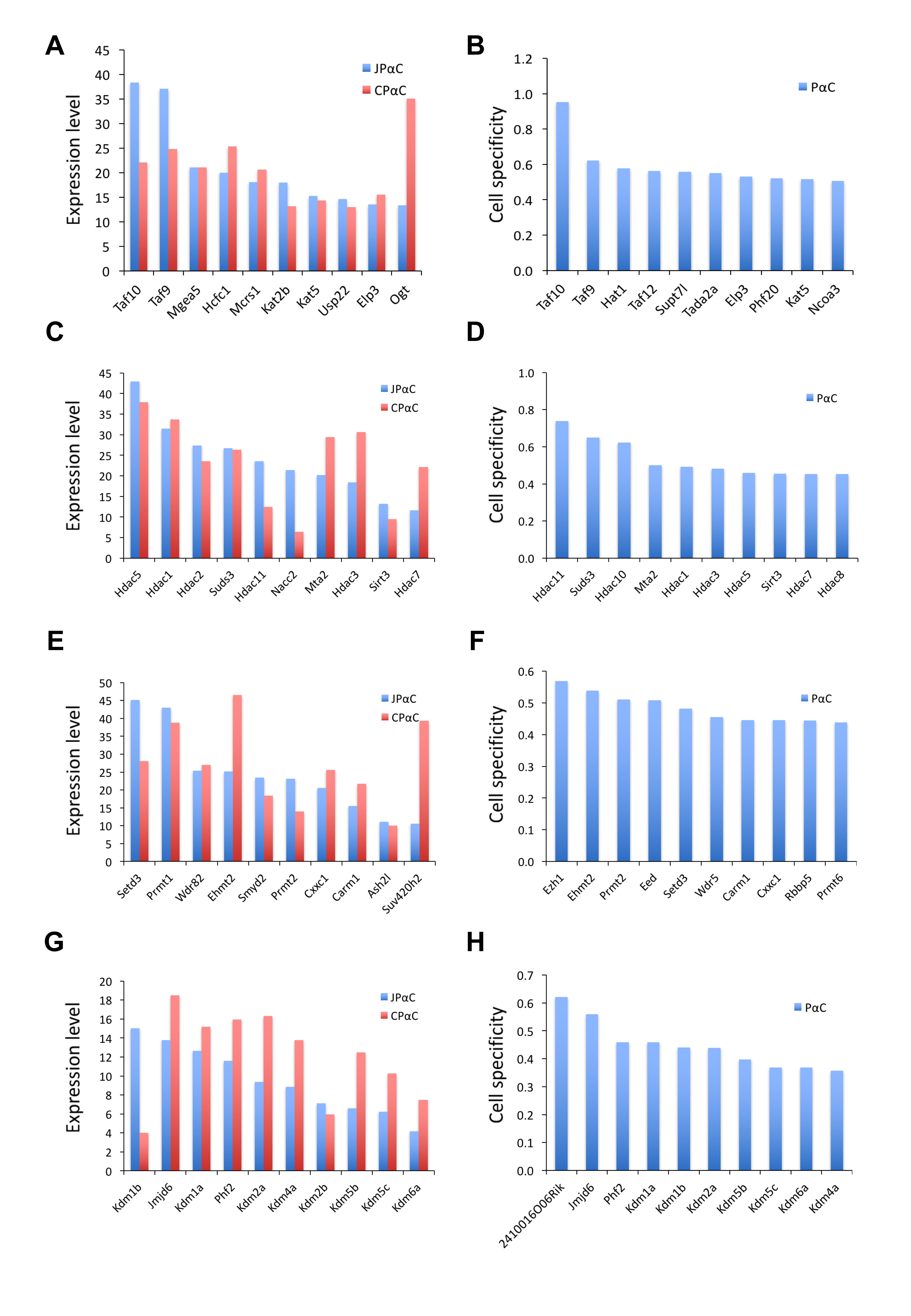

Supplement: S7 Fig — (A) Histone acetyltransferases enriched in JPαC and CPαC. (B) PαC-specific histone acetyltransferases. (C) Histone deacetylases enriched in JPαC and CPαC. (D) ICC-specific histone deacetylases. (E) Histone methyltransferases enriched in JPαC and CPαC. (F) PαC-specific histone methyltransferases. (G) Histone demethylases enriched in JPαC and CPαC. (H) PαC-specific histone demethylases. Cell specificity was determined by comparative analysis of gene expression profiles among PαC, SMC, and ICC. (TIF) [file pone.0182265.s007.tif]

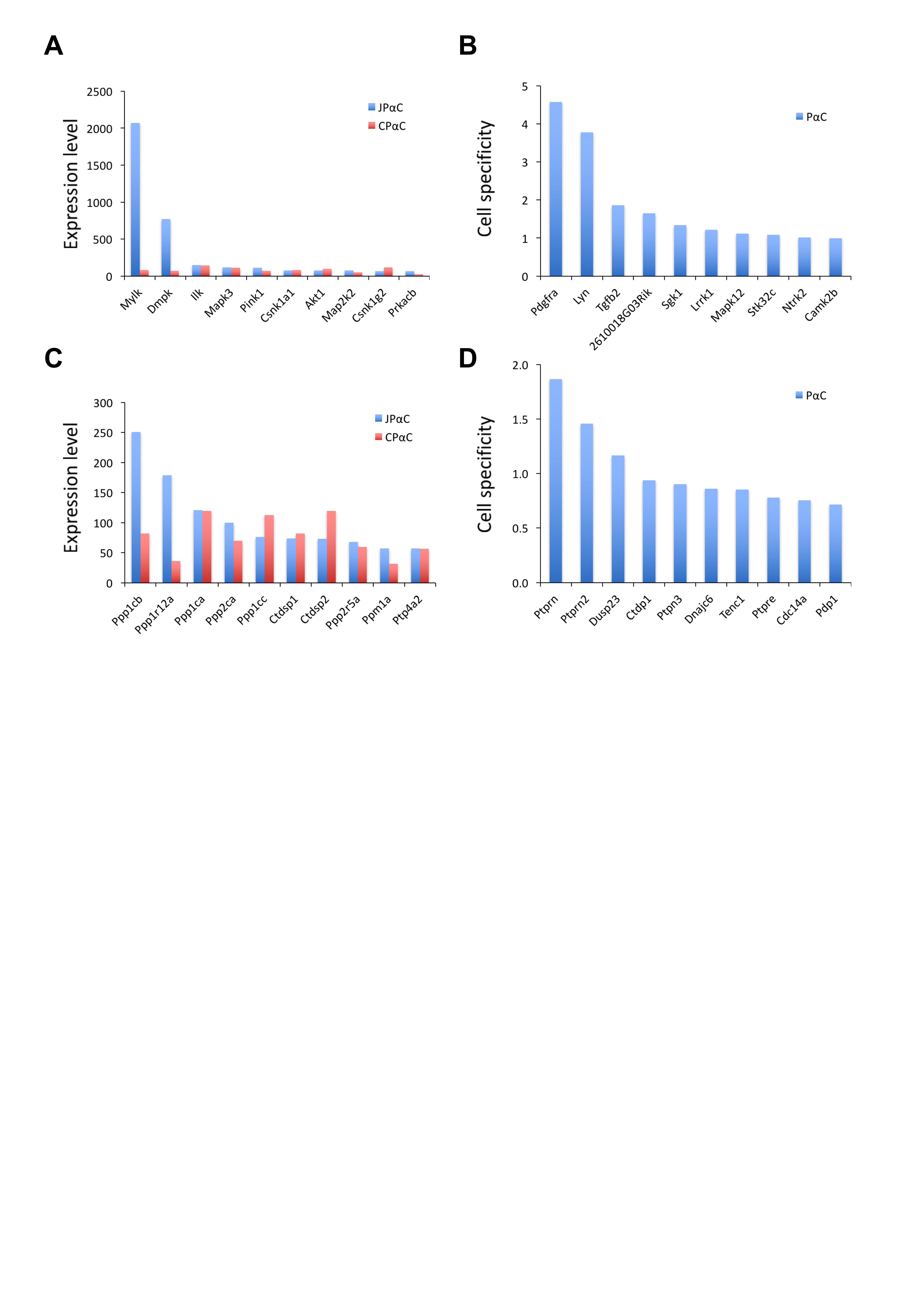

Supplement: S8 Fig — (A) Protein kinases enriched in JPαC and CPαC. (B) PαC-specific protein kinases. (C) Phosphatases enriched in JPαC and CPαC. (D) PαC-specific phosphatases. Cell specificity was determined by comparative analysis of gene expression profiles among PαC, SMC, and ICC. (TIF) [file pone.0182265.s008.tif]
