# Supplementary material for: Transcriptome analysis of PDGFRα+ cells identifies T-type Ca2+ channel CACNA1G as a new pathological marker for PDGFRα+ cell hyperplasia
Source: PLoS One. 2017 Aug 14;12(8):e0182265. doi: 10.1371/journal.pone.0182265 (PMC5555714; doi:10.1371/journal.pone.0182265)
Supplement: S2 Fig — The open reading frame was identified for each transcriptional variant, and all predicted amino acid sequences were aligned. Six transmembrane helices (S1–S6) in four homologous domains (I-IV) are shown. Colors on amino acid sequence show distinct regions and segments. Green are start codons found in differentially spliced variants. Purple are positively charged residues in S4 voltage sensing segments. Red are missing or inserted peptides from differentially spliced exons. (DOCX) [file pone.0182265.s002.docx]

966 MDEEEDGAGAEESGQPRSFTQLNDLSGAGGRQGPGSTEKDPGSADSEAEGLPYPALAPVV 60

785 MDEEEDGAGAEESGQPRSFTQLNDLSGAGGRQGPGSTEKDPGSADSEAEGLPYPALAPVV 60

784 MDEEEDGAGAEESGQPRSFTQLNDLSGAGGRQGPGSTEKDPGSADSEAEGLPYPALAPVV 60

783 MDEEEDGAGAEESGQPRSFTQLNDLSGAGGRQGPGSTEKDPGSADSEAEGLPYPALAPVV 60

176 MDEEEDGAGAEESGQPRSFTQLNDLSGAGGRQGPGSTEKDPGSADSEAEGLPYPALAPVV 60

---------**IS1**--------- -

966 FFYLSQDSRPRSWCLRTVCNPWFERVSMLVILLNCVTLGMFRPCEDIACDSQRCRILQAF 120

785 FFYLSQDSRPRSWCLRTVCNPWFERVSMLVILLNCVTLGMFRPCEDIACDSQRCRILQAF 120

784 FFYLSQDSRPRSWCLRTVCNPWFERVSMLVILLNCVTLGMFRPCEDIACDSQRCRILQAF 120

783 FFYLSQDSRPRSWCLRTVCNPWFERVSMLVILLNCVTLGMFRPCEDIACDSQRCRILQAF 120

176 FFYLSQDSRPRSWCLRTVCNPWFERVSMLVILLNCVTLGMFRPCEDIACDSQRCRILQAF 120

---------**IS2-**--------- --------**IS3**--------- -----

966 DDFIFAFFAVEMVVKMVALGIFGKKCYLGDTWNRLDFFIVIAGMLEYSLDLQNVSFSAVR 180

785 DDFIFAFFAVEMVVKMVALGIFGKKCYLGDTWNRLDFFIVIAGMLEYSLDLQNVSFSAVR 180

784 DDFIFAFFAVEMVVKMVALGIFGKKCYLGDTWNRLDFFIVIAGMLEYSLDLQNVSFSAVR 180

783 DDFIFAFFAVEMVVKMVALGIFGKKCYLGDTWNRLDFFIVIAGMLEYSLDLQNVSFSAVR 180

176 DDFIFAFFAVEMVVKMVALGIFGKKCYLGDTWNRLDFFIVIAGMLEYSLDLQNVSFSAVR 180

---**IS4**-------- ---------**IS5**---------

966 TVRVLRPLRAINRVPSMRILVTLLLDTLPMLGNVLLLCFFVFFIFGIVGVQLWAGLLRNR 240

785 TVRVLRPLRAINRVPSMRILVTLLLDTLPMLGNVLLLCFFVFFIFGIVGVQLWAGLLRNR 240

784 TVRVLRPLRAINRVPSMRILVTLLLDTLPMLGNVLLLCFFVFFIFGIVGVQLWAGLLRNR 240

783 TVRVLRPLRAINRVPSMRILVTLLLDTLPMLGNVLLLCFFVFFIFGIVGVQLWAGLLRNR 240

176 TVRVLRPLRAINRVPSMRILVTLLLDTLPMLGNVLLLCFFVFFIFGIVGVQLWAGLLRNR 240

966 CFLPENFSLPLSVDLEPYYQTENEDESPFICSQPRENGMRSCRSVPTLRGEGGGGPPCGL 300

785 CFLPENFSLPLSVDLEPYYQTENEDESPFICSQPRENGMRSCRSVPTLRGEGGGGPPCGL 300

784 CFLPENFSLPLSVDLEPYYQTENEDESPFICSQPRENGMRSCRSVPTLRGEGGGGPPCGL 300

783 CFLPENFSLPLSVDLEPYYQTENEDESPFICSQPRENGMRSCRSVPTLRGEGGGGPPCGL 300

176 CFLPENFSLPLSVDLEPYYQTENEDESPFICSQPRENGMRSCRSVPTLRGEGGGGPPCGL 300

966 DYEAYNSSSNTTCVNWNQYYTNCSAGEHNPFKGAINFDNIGYAWIAIFQVITLEGWVDIM 360

785 DYEAYNSSSNTTCVNWNQYYTNCSAGEHNPFKGAINFDNIGYAWIAIFQVITLEGWVDIM 360

784 DYEAYNSSSNTTCVNWNQYYTNCSAGEHNPFKGAINFDNIGYAWIAIFQVITLEGWVDIM 360

783 DYEAYNSSSNTTCVNWNQYYTNCSAGEHNPFKGAINFDNIGYAWIAIFQVITLEGWVDIM 360

176 DYEAYNSSSNTTCVNWNQYYTNCSAGEHNPFKGAINFDNIGYAWIAIFQVITLEGWVDIM 360

-----------**IS6**-----------

966 YFVMDAHSFYNFIYFILLIIVGSFFMINLCLVVIATQFSETKQRESQLMREQRVRFLSNA 420

785 YFVMDAHSFYNFIYFILLII-----------------FSETKQRESQLMREQRVRFLSNA 403

784 YFVMDAHSFYNFIYFILLIIVGSFFMINLCLVVIATQFSETKQRESQLMREQRVRFLSNA 420

783 YFVMDAHSFYNFIYFILLIIVGSFFMINLCLVVIATQFSETKQRESQLMREQRVRFLSNA 420

176 YFVMDAHSFYNFIYFILLII-----------------FSETKQRESQLMREQRVRFLSNA 403

966 STLASFSEPGSCYEELLKYLVYILRKAARRLAQVSRAVGVRAGLLSSPVARGGQEPQPSG 480

785 STLASFSEPGSCYEELLKYLVYILRKAARRLAQVSRAVGVRAGLLSSPVARGGQEPQPSG 463

784 STLASFSEPGSCYEELLKYLVYILRKAARRLAQVSRAVGVRAGLLSSPVARGGQEPQPSG 480

783 STLASFSEPGSCYEELLKYLVYILRKAARRLAQVSRAVGVRAGLLSSPVARGGQEPQPSG 480

176 STLASFSEPGSCYEELLKYLVYILRKAARRLAQVSRAVGVRAGLLSSPVARGGQEPQPSG 463

966 SCSRSHRRLSVHHLVHHHHHHHHHYHLGNGTLRVPRASPEIQDRDANGSRWLMLPPPSTP 540

785 SCSRSHRRLSVHHLVHHHHHHHHHYHLGNGTLRVPRASPEIQDRDANGSRWLMLPPPSTP 523

784 SCSRSHRRLSVHHLVHHHHHHHHHYHLGNGTLRVPRASPEIQDRDANGSRWLMLPPPSTP 540

783 SCSRSHRRLSVHHLVHHHHHHHHHYHLGNGTLRVPRASPEIQDRDANGSRWLMLPPPSTP 540

176 SCSRSHRRLSVHHLVHHHHHHHHHYHLGNGTLRVPRASPEIQDRDANGSRWLMLPPPSTP 523

966 TPSGGPPRGAESVHSFYHADCHLEPVRCQAPPPRSPSEASGRTVGSGKVYPTVHTSPPPE 600

785 TPSGGPPRGAESVHSFYHADCHLEPVRCQAPPPRSPSEASGRTVGSGKVYPTVHTSPPPE 583

784 TPSGGPPRGAESVHSFYHADCHLEPVRCQAPPPRSPSEASGRTVGSGKVYPTVHTSPPPE 600

783 TPSGGPPRGAESVHSFYHADCHLEPVRCQAPPPRSPSEASGRTVGSGKVYPTVHTSPPPE 600

176 TPSGGPPRGAESVHSFYHADCHLEPVRCQAPPPRSPSEASGRTVGSGKVYPTVHTSPPPE 583

966 MLKDKALVEVAPSPGPPTLTSFNIPPGPFSSMHKLLETQSTGACHSSCKISSPCSKADSG 660

785 MLKDKALVEVAPSPGPPTLTSFNIPPGPFSSMHKLLETQSTGACHSSCKISSPCSKADSG 643

784 MLKDKALVEVAPSPGPPTLTSFNIPPGPFSSMHKLLETQSTGACHSSCKISSPCSKADSG 660

783 MLKDKALVEVAPSPGPPTLTSFNIPPGPFSSMHKLLETQSTGACHSSCKISSPCSKADSG 660

176 MLKDKALVEVAPSPGPPTLTSFNIPPGPFSSMHKLLETQSTGACHSSCKISSPCSKADSG 643

966 ACGPDSCPYCARTGAGEPESADHEMPDSDSEAVYEFTQDAQHSDLRDPHRRRRPSLGPDA 720

785 ACGPDSCPYCARTGAGEPESADHEMPDSDSEAVYEFTQDAQHSDLRDPHRRRRPSLGPDA 703

784 ACGPDSCPYCARTGAGEPESADHEMPDSDSEAVYEFTQDAQHSDLRDPHRRRRPSLGPDA 720

783 ACGPDSCPYCARTGAGEPESADHEMPDSDSEAVYEFTQDAQHSDLRDPHRRRRPSLGPDA 720

176 ACGPDSCPYCARTGAGEPESADHEMPDSDSEAVYEFTQDAQHSDLRDPHRRRRPSLGPDA 703

---------**IIS1**-------- ---

966 EPSSVLAFWRLICDTFRKIVDSKYFGRGIMIAILVNTLSMGIEYHEQPEELTNALEISNI 780

785 EPSSVLAFWRLICDTFRKIVDSKYFGRGIMIAILVNTLSMGIEYHEQPEELTNALEISNI 763

784 EPSSVLAFWRLICDTFRKIVDSKYFGRGIMIAILVNTLSMGIEYHEQPEELTNALEISNI 780

783 EPSSVLAFWRLICDTFRKIVDSKYFGRGIMIAILVNTLSMGIEYHEQPEELTNALEISNI 780

176 EPSSVLAFWRLICDTFRKIVDSKYFGRGIMIAILVNTLSMGIEYHEQPEELTNALEISNI 763

-------**IIS2**-------- --------**IIS3**------- -------

465 ----------------------------------------------------------MR 2

966 VFTSLFALEMLLKLLVYGPFGYIKNPYNIFDGVIVVISVWEIVGQQGGGLSVLRTFRLMR 840

785 VFTSLFALEMLLKLLVYGPFGYIKNPYNIFDGVIVVISVWEIVGQQGGGLSVLRTFRLMR 823

784 VFTSLFALEMLLKLLVYGPFGYIKNPYNIFDGVIVVISVWEIVGQQGGGLSVLRTFRLMR 840

783 VFTSLFALEMLLKLLVYGPFGYIKNPYNIFDGVIVVISVWEIVGQQGGGLSVLRTFRLMR 840

176 VFTSLFALEMLLKLLVYGPFGYIKNPYNIFDGVIVVISVWEIVGQQGGGLSVLRTFRLMR 823

---**IIS4--**------- ---------**IIS5**--------

465 VLKLVRFLPALQRQLVVLMKTMDNVATFCMLLMLFIFIFSILGMHLFGCKFASERDGDTL 62

966 VLKLVRFLPALQRQLVVLMKTMDNVATFCMLLMLFIFIFSILGMHLFGCKFASERDGDTL 900

785 VLKLVRFLPALQRQLVVLMKTMDNVATFCMLLMLFIFIFSILGMHLFGCKFASERDGDTL 883

784 VLKLVRFLPALQRQLVVLMKTMDNVATFCMLLMLFIFIFSILGMHLFGCKFASERDGDTL 900

783 VLKLVRFLPALQRQLVVLMKTMDNVATFCMLLMLFIFIFSILGMHLFGCKFASERDGDTL 900

176 VLKLVRFLPALQRQLVVLMKTMDNVATFCMLLMLFIFIFSILGMHLFGCKFASERDGDTL 883

-----------**IIS6**------

465 PDRKNFDSLLWAIVTVFQILTQEDWNKVLYNGMASTSSWAALYFIALMTFGNYVLFNLLV 122

966 PDRKNFDSLLWAIVTVFQILTQEDWNKVLYNGMASTSSWAALYFIALMTFGNYVLFNLLV 960

785 PDRKNFDSLLWAIVTVFQILTQEDWNKVLYNGMASTSSWAALYFIALMTFGNYVLFNLLV 943

784 PDRKNFDSLLWAIVTVFQILTQEDWNKVLYNGMASTSSWAALYFIALMTFGNYVLFNLLV 960

783 PDRKNFDSLLWAIVTVFQILTQEDWNKVLYNGMASTSSWAALYFIALMTFGNYVLFNLLV 960

176 PDRKNFDSLLWAIVTVFQILTQEDWNKVLYNGMASTSSWAALYFIALMTFGNYVLFNLLV 943

----

465 AILVEGFQAEEIGKREDTSGQLSCIQLPVNSQGGDATKSESEPDFFSPSVDGDGDRKKRL 182

966 AILVEGFQAEEIGKREDTSGQLSCIQLPVNSQGGDATKSESEPDFFSPSVDGDGDRKKRL 1020

785 AILVEGFQAE-----------------------GDATKSESEPDFFSPSVDGDGDRKKRL 980

784 AILVEGFQAE-----------------------GDATKSESEPDFFSPSVDGDGDRKKRL 997

783 AILVEGFQAE-----------------------GDATKSESEPDFFSPSVDGDGDRKKRL 997

176 AILVEGFQAEEIGKREDTSGQLSCIQLPVNSQGGDATKSESEPDFFSPSVDGDGDRKKRL 1003

465 ALVALGEHSELRKSLLPPLIIHTAATPMSLPKSSSTGVGEALGSGSRRTSSSGSAEPGTA 242

966 ALVALGEHSELRKSLLPPLIIHTAATPMSLPKSSSTGVGEALGSGSRRTSSSGSAEPGTA 1080

464 ---------------------------MSLPKSSSTGVGEALGSGSRRTSSSGSAEPGTA 33

785 ALVALGEHSELRKSLLPPLIIHTAATPMSLPKSSSTGVGEALGSGSRRTSSSGSAEPGTA 1040

784 ALVALGEHSELRKSLLPPLIIHTAATPMSLPKSSSTGVGEALGSGSRRTSSSGSAEPGTA 1057

783 ALVALGEHSELRKSLLPPLIIHTAATPMSLPKSSSTGVGEALGSGSRRTSSSGSAEPGTA 1057

176 ALVALGEHSELRKSLLPPLIIHTAATPMSLPKSSSTGVGEALGSGSRRTSSSGSAEPGTA 1063

465 HHEMKSPPSARSSPHSPWSAASSWTSRRSSRNSLGRAPSLKRRSPSGERRSLLSGEGQES 302

966 HHEMKSPPSARSSPHSPWSAASSWTSRRSSRNSLGRAPSLKRRSPSGERRSLLSGEGQES 1140

464 HHEMKSPPSARSSPHSPWSAASSWTSRRSSRNSLGRAPSLKRRSPSGERRSLLSGEGQES 93

785 HHEMKSPPSARSSPHSPWSAASSWTSRRSSRNSLGRAPSLKRRSPSGERRSLLSGEGQES 1100

784 HHEMKSPPSARSSPHSPWSAASSWTSRRSSRNSLGRAPSLKRRSPSGERRSLLSGEGQES 1117

783 HHEMKSPPSARSSPHSPWSAASSWTSRRSSRNSLGRAPSLKRRSPSGERRSLLSGEGQES 1117

176 HHEMKSPPSARSSPHSPWSAASSWTSRRSSRNSLGRAPSLKRRSPSGERRSLLSGEGQES 1123

465 QDEEESSEEDRASPAGSDHRHRGSLEREAKSSFDLPDTLQVPGLHRTASGRSSASEHQDC 362

966 QDEEESSEEDRASPAGSDHRHRGSLEREAKSSFDLPDTLQVPGLHRTASGRSSASEHQDC 1200

464 QDEEESSEEDRASPAGSDHRHRGSLEREAKSSFDLPDTLQVPGLHRTASGRSSASEHQDC 153

785 QDEEESSEEDRASPAGSDHRHRGSLEREAKSSFDLPDTLQVPGLHRTASGRSSASEHQDC 1160

784 QDEEESSEEDRASPAGSDHRHRGSLEREAKSSFDLPDTLQVPGLHRTASGRSSASEHQDC 1177

783 QDEEESSEEDRASPAGSDHRHRGSLEREAKSSFDLPDTLQVPGLHRTASGRSSASEHQDC 1177

176 QDEEESSEEDRASPAGSDHRHRGSLEREAKSSFDLPDTLQVPGLHRTASGRSSASEHQDC 1183

465 NGKSASGRLARTLRADDPPLDGDDGDDEGNLSKGERLRAWVRARLPACCRERDSWSAYIF 422

966 NGKSASGRLARTLRADDPPLDGDDGDDEGNLSKGERLRAWVRARLPACCRERDSWSAYIF 1260

464 NGKSASGRLARTLRADDPPLDGDDGDDEGNLSKGERLRAWVRARLPACCRERDSWSAYIF 213

785 NGKSASGRLARTLRADDPPLDGDDGDDEGNLSKGERLRAWVRARLPACCRERDSWSAYIF 1220

784 NGKSASGRLARTLRADDPPLDGDDGDDEGNLSKGERLRAWVRARLPACCRERDSWSAYIF 1237

783 NGKSASGRLARTLRADDPPLDGDDGDDEGNLSKGERLRAWVRARLPACCRERDSWSAYIF 1237

176 NGKSASGRLARTLRADDPPLDGDDGDDEGNLSKGERLRAWVRARLPACCRERDSWSAYIF 1243

---------**IIIS1**--------- -----

465 PPQSRFRLLCHRIITHKMFDHVVLVIIFLNCITIAMERPKIDPHSAERIFLTLSNYIFTA 482

966 PPQSRFRLLCHRIITHKMFDHVVLVIIFLNCITIAMERPKIDPHSAERIFLTLSNYIFTA 1320

464 PPQSRFRLLCHRIITHKMFDHVVLVIIFLNCITIAMERPKIDPHSAERIFLTLSNYIFTA 273

785 PPQSRFRLLCHRIITHKMFDHVVLVIIFLNCITIAMERPKIDPHSAERIFLTLSNYIFTA 1280

784 PPQSRFRLLCHRIITHKMFDHVVLVIIFLNCITIAMERPKIDPHSAERIFLTLSNYIFTA 1297

783 PPQSRFRLLCHRIITHKMFDHVVLVIIFLNCITIAMERPKIDPHSAERIFLTLSNYIFTA 1297

176 PPQSRFRLLCHRIITHKMFDHVVLVIIFLNCITIAMERPKIDPHSAERIFLTLSNYIFTA 1303

--**IIIS2**-------- -------**IIIS3**-------- -

465 VFLAEMTVKVVALGWCFGEQAYLRSSWNVLDGLLVLISVIDILVSMVSDSGTKILGMLRV 542

966 VFLAEMTVKVVALGWCFGEQAYLRSSWNVLDGLLVLISVIDILVSMVSDSGTKILGMLRV 1380

464 VFLAEMTVKVVALGWCFGEQAYLRSSWNVLDGLLVLISVIDILVSMVSDSGTKILGMLRV 333

785 VFLAEMTVKVVALGWCFGEQAYLRSSWNVLDGLLVLISVIDILVSMVSDSGTKILGMLRV 1340

784 VFLAEMTVKVVALGWCFGEQAYLRSSWNVLDGLLVLISVIDILVSMVSDSGTKILGMLRV 1357

783 VFLAEMTVKVVALGWCFGEQAYLRSSWNVLDGLLVLISVIDILVSMVSDSGTKILGMLRV 1357

176 VFLAEMTVKVVALGWCFGEQAYLRSSWNVLDGLLVLISVIDILVSMVSDSGTKILGMLRV 1363

-------**IIIS4**--------- ----------**IIIS5**---------

465 LRLLRTLRPLRVISRAQGLKLVVETLMSSLKPIGNIVVICCAFFIIFGILGVQLFKGKFF 602

966 LRLLRTLRPLRVISRAQGLKLVVETLMSSLKPIGNIVVICCAFFIIFGILGVQLFKGKFF 1440

464 LRLLRTLRPLRVISRAQGLKLVVETLMSSLKPIGNIVVICCAFFIIFGILGVQLFKGKFF 393

785 LRLLRTLRPLRVISRAQGLKLVVETLMSSLKPIGNIVVICCAFFIIFGILGVQLFKGKFF 1400

784 LRLLRTLRPLRVISRAQGLKLVVETLMSSLKPIGNIVVICCAFFIIFGILGVQLFKGKFF 1417

783 LRLLRTLRPLRVISRAQGLKLVVETLMSSLKPIGNIVVICCAFFIIFGILGVQLFKGKFF 1417

176 LRLLRTLRPLRVISRAQGLKLVVETLMSSLKPIGNIVVICCAFFIIFGILGVQLFKGKFF 1423

465 VCQGEDTRNITNKSDCAEASYRWVRHKYNFDNLGQALMSLFVLASKDGWVDIMYDGLDAV 662

966 VCQGEDTRNITNKSDCAEASYRWVRHKYNFDNLGQALMSLFVLASKDGWVDIMYDGLDAV 1500

464 VCQGEDTRNITNKSDCAEASYRWVRHKYNFDNLGQALMSLFVLASKDGWVDIMYDGLDAV 453

785 VCQGEDTRNITNKSDCAEASYRWVRHKYNFDNLGQALMSLFVLASKDGWVDIMYDGLDAV 1460

784 VCQGEDTRNITNKSDCAEASYRWVRHKYNFDNLGQALMSLFVLASKDGWVDIMYDGLDAV 1477

783 VCQGEDTRNITNKSDCAEASYRWVRHKYNFDNLGQALMSLFVLASKDGWVDIMYDGLDAV 1477

965 -------------------------------------MSLFVLASKDGWVDIMYDGLDAV 23

176 VCQGEDTRNITNKSDCAEASYRWVRHKYNFDNLGQALMSLFVLASKDGWVDIMYDGLDAV 1483

-----------**IIIS6**----------

465 GVDQQPIMNHNPWMLLYFISFLLIVAFFVLNMFVGVVVENFHKCRQHQEEEEARRREEKR 722

966 GVDQQPIMNHNPWMLLYFISFLLIVAFFVLNMFVGVVVENFHKCRQHQEEEEARRREEKR 1560

464 GVDQQPIMNHNPWMLLYFISFLLIVAFFVLNMFVGVVVENFHKCRQHQEEEEARRREEKR 513

785 GVDQQPIMNHNPWMLLYFISFLLIVAFFVLNMFVGVVVENFHKCRQHQEEEEARRREEKR 1520

784 GVDQQPIMNHNPWMLLYFISFLLIVAFFVLNMFVGVVVENFHKCRQHQEEEEARRREEKR 1537

783 GVDQQPIMNHNPWMLLYFISFLLIVAFFVLNMFVGVVVENFHKCRQHQEEEEARRREEKR 1537

965 GVDQQPIMNHNPWMLLYFISFLLIVAFFVLNMFVGVVVENFHKCRQHQEEEEARRREEKR 83

176 GVDQQPIMNHNPWMLLYFISFLLIVAFFVLNMFVGVVVENFHKCRQHQEEEEARRREEKR 1543

--

465 LKRLEKKRR-------NLMLDDVIASGSSASAASEAQCKPYYSDYSRFRLLVHHLCTSHY 775

966 LKRLEKKRR-------NLMLDDVIASGSSASAASEAQCKPYYSDYSRFRLLVHHLCTSHY 1613

464 LKRLEKKRR-------NLMLDDVIASGSSASAASEAQCKPYYSDYSRFRLLVHHLCTSHY 566

785 LKRLEKKRR-------NLMLDDVIASGSSASAASEAQCKPYYSDYSRFRLLVHHLCTSHY 1573

784 LKRLEKKRR-------NLMLDDVIASGSSASAASEAQCKPYYSDYSRFRLLVHHLCTSHY 1590

783 LKRLEKKRRSKEKQMADLMLDDVIASGSSASAASEAQCKPYYSDYSRFRLLVHHLCTSHY 1597

965 LKRLEKKRRSKEKQMADLMLDDVIASGSSASAASEAQCKPYYSDYSRFRLLVHHLCTSHY 143

865 ---------MSCFVLLDLMLDDVIASGSSASAASEAQCKPYYSDYSRFRLLVHHLCTSHY 51

113 ------------------MLDDVIASGSSASAASEAQCKPYYSDYSRFRLLVHHLCTSHY 42

176 LKRLEKKRRSKEKQMAGR------------------------------------------ 1561

------**IVS1**--------- ---------**IVS2**---------

465 LDLFITGVIGLNVVTMAMEHYQQPQILDEALKICNYIFTVIFVLESVFKLVAFGFRRFFQ 835

966 LDLFITGVIGLNVVTMAMEHYQQPQILDEALKICNYIFTVIFVLESVFKLVAFGFRRFFQ 1673

464 LDLFITGVIGLNVVTMAMEHYQQPQILDEALKICNYIFTVIFVLESVFKLVAFGFRRFFQ 626

785 LDLFITGVIGLNVVTMAMEHYQQPQILDEALKICNYIFTVIFVLESVFKLVAFGFRRFFQ 1633

784 LDLFITGVIGLNVVTMAMEHYQQPQILDEALKICNYIFTVIFVLESVFKLVAFGFRRFFQ 1650

783 LDLFITGVIGLNVVTMAMEHYQQPQILDEALKICNYIFTVIFVLESVFKLVAFGFRRFFQ 1657

965 LDLFITGVIGLNVVTMAMEHYQQPQILDEALKICNYIFTVIFVLESVFKLVAFGFRRFFQ 203

865 LDLFITGVIGLNVVTMAMEHYQQPQILDEALKICNYIFTVIFVLESVFKLVAFGFRRFFQ 111

113 LDLFITGVIGLNVVTMAMEHYQQPQILDEALKICNYIFTVIFVLESVFKLVAFGFRRFFQ 102

--------**IVS3**------- ----------**IVS4**----------

465 DRWNQLDLAIVLLSIMGITLEEIEVNASLPINPTIIRIMRVLRIARVLKLLKMAVGMRAL 895

966 DRWNQLDLAIVLLSIMGITLEEIEVNASLPINPTIIRIMRVLRIARVLKLLKMAVGMRAL 1733

464 DRWNQLDLAIVLLSIMGITLEEIEVNASLPINPTIIRIMRVLRIARVLKLLKMAVGMRAL 686

785 DRWNQLDLAIVLLSIMGITLEEIEVNASLPINPTIIRIMRVLRIARVLKLLKMAVGMRAL 1693

784 DRWNQLDLAIVLLSIMGITLEEIEVNASLPINPTIIRIMRVLRIARVLKLLKMAVGMRAL 1710

783 DRWNQLDLAIVLLSIMGITLEEIEVNASLPINPTIIRIMRVLRIARVLKLLKMAVGMRAL 1717

965 DRWNQLDLAIVLLSIMGITLEEIEVNASLPINPTIIRIMRVLRIARVLKLLKMAVGMRAL 263

865 DRWNQLDLAIVLLSIMGITLEEIEVNASLPINPTIIRIMRVLRIARVLKLLKMAVGMRAL 171

113 DRWNQLDLAIVLLSIMGITLEEIEVNASLPINPTIIRIMRVLRIAR-------------- 148

---------**IVS5**--------

465 LDTVMQALPQVGNLGLLFMLLFFIFAALGVELFGDLECDETHPCEGLGRHATFRNFGMAF 955

463 ---------------------------------------------------------MAF 3

966 LDTVMQALPQVGNLGLLFMLLFFIFAALGVELFGDLECDETHPCEGLGRHATFRNFGMAF 1793

464 LDTVMQALPQVGNLGLLFMLLFFIFAALGVELFGDLECDETHPCEGLGRHATFRNFGMAF 746

785 LDTVMQALPQVGNLGLLFMLLFFIFAALGVELFGDLECDETHPCEGLGRHATFRNFGMAF 1753

784 LDTVMQALPQVGNLGLLFMLLFFIFAALGVELFGDLECDETHPCEGLGRHATFRNFGMAF 1770

783 LDTVMQALPQVGNLGLLFMLLFFIFAALGVELFGDLECDETHPCEGLGRHATFRNFGMAF 1777

965 LDTVMQALPQVGNLGLLFMLLFFIFAALGVELFGDLECDETHPCEGLGRHATFRNFGMAF 323

865 LDTVMQALPQVGNLGLLFMLLFFIFAALGVELFGDLECDETHPCEGLGRHATFRNFGMAF 231

113 --------------------------ALGVELFGDLECDETHPCEGLGRHATFRNFGMAF 182

------------**IVS6**----------

465 LTLFRVSTGDNWNGIMKDTLRDCDQESTCYNTVISPIYFVSFVLTAQFVLVNVVIAVLMK 1015

463 LTLFRVSTGDNWNGIMKDTLRDCDQESTCYNTVISPIYFVSFVLTAQFVLVNVVIAVLMK 63

966 LTLFRVSTGDNWNGIMKDTLRDCDQESTCYNTVISPIYFVSFVLTAQFVLVNVVIAVLMK 1853

464 LTLFRVSTGDNWNGIMKDTLRDCDQESTCYNTVISPIYFVSFVLTAQFVLVNVVIAVLMK 806

785 LTLFRVSTGDNWNGIMKDTLRDCDQESTCYNTVISPIYFVSFVLTAQFVLVNVVIAVLMK 1813

784 LTLFRVSTGDNWNGIMKDTLRDCDQESTCYNTVISPIYFVSFVLTAQFVLVNVVIAVLMK 1830

783 LTLFRVSTGDNWNGIMKDTLRDCDQESTCYNTVISPIYFVSFVLTAQFVLVNVVIAVLMK 1837

965 LTLFRVSTGDNWNGIMKDTLRDCDQESTCYNTVISPIYFVSFVLTAQFVLVNVVIAVLMK 383

865 LTLFRVSTGDNWNGIMKDTLRDCDQESTCYNTVISPIYFVSFVLTAQFVLVNVVIAVLMK 291

113 LTLFRVSTGDNWNGIMKDTLRDCDQESTCYNTVISPIYFVSFVLTAQFVLVNVVIAVLMK 242

--

465 HLEESNKEAKEEAELEAELELEMKTLSPQPHSPLGSPFLWPGVEGVNSPDSPKPGAPHTT 1075

463 HLEESNKEAKEEAELEAELELEMKTLSPQPHSPLGSPFLWPGVEGVNSPDSPKPGAPHTT 123

966 HLEESNKEAKEEAELEAELELEMKTLSPQPHSPLGSPFLWPGVEGVNSPDSPKPGAPHTT 1913

464 HLEESNKEAKEEAELEAELELEMKTLSPQPHSPLGSPFLWPGVEGVNSPDSPKPGAPHTT 866

785 HLEESNKEAKEEAELEAELELEMKTLSPQPHSPLGSPFLWPGVEGVNSPDSPKPGAPHTT 1873

784 HLEESNKEAKEEAELEAELELEMKTLSPQPHSPLGSPFLWPGVEGVNSPDSPKPGAPHTT 1890

783 HLEESNKEAKEEAELEAELELEMKTLSPQPHSPLGSPFLWPGVEGVNSPDSPKPGAPHTT 1897

965 HLEESNKEAKEEAELEAELELEMKTLSPQPHSPLGSPFLWPGVEGVNSPDSPKPGAPHTT 443

865 HLEESNKEAKEEAELEAELELEMKTLSPQPHSPLGSPFLWPGVEGVNSPDSPKPGAPHTT 351

113 HLEESNKEAKEEAELEAELELEMKTLSPQPHSPLGSPFLWPGVEGVNSPDSPKPGAPHTT 302

465 AHIGAASSGFSLEHPT-------------------------------------------- 1091

463 AHIGAASSGFSLEHPT-------------------------------------------- 139

966 AHIGAASSGFSLEHPT-------------------------------------------- 1929

464 AHIGAASSGFSLEHPT-------------------------------------------- 882

785 AHIGAASSGFSLEHPT-------------------------------------------- 1889

784 AHIGAASSGFSLEHPT-------------------------------------------- 1906

783 AHIGAASSGFSLEHPT-------------------------------------------- 1913

965 AHIGAASSGFSLEHPT-------------------------------------------- 459

865 AHIGAASSGFSLEHPT-------------------------------------------- 367

113 AHIGAASSGFSLEHPTDRQLFDTISLLIQGSLEGELKLMDELAGPGGQPSAFPSAPSPGD 362

465 ----MVPHTEEGPVPLGPDLLTVRKSGVSRTHSLPNDSYMCRNGSTAERSLGHRGWGLPK 1147

463 ----MVPHTEEGPVPLGPDLLTVRKSGVSRTHSLPNDSYMCRNGSTAERSLGHRGWGLPK 195

966 ----MVPHTEEGPVPLGPDLLTVRKSGVSRTHSLPNDSYMCRNGSTAERSLGHRGWGLPK 1985

464 ----MVPHTEEGPVPLGPDLLTVRKSGVSRTHSLPNDSYMCRNGSTAERSLGHRGWGLPK 938

785 ----MVPHTEEGPVPLGPDLLTVRKSGVSRTHSLPNDSYMCRNGSTAERSLGHRGWGLPK 1945

784 ----MVPHTEEGPVPLGPDLLTVRKSGVSRTHSLPNDSYMCRNGSTAERSLGHRGWGLPK 1962

783 ----MVPHTEEGPVPLGPDLLTVRKSGVSRTHSLPNDSYMCRNGSTAERSLGHRGWGLPK 1969

965 ----MVPHTEEGPVPLGPDLLTVRKSGVSRTHSLPNDSYMCRNGSTAERSLGHRGWGLPK 515

865 ----MVPHTEEGPVPLGPDLLTVRKSGVSRTHSLPNDSYMCRNGSTAERSLGHRGWGLPK 423

113 SDPQMVPHTEEGPVPLGPDLLTVRKSGVSRTHSLPNDSYMCRNGSTAERSLGHRGWGLPK 422

465 AQSGSILSVHSQPADTSCILQLPKDAHYLLQPHGAPTWGAIPKLPPPGRSPLAQRPLRRQ 1207

463 AQSGSILSVHSQPADTSCILQLPKDAHYLLQPHGAPTWGAIPKLPPPGRSPLAQRPLRRQ 255

966 AQSGSILSVHSQPADTSCILQLPKDAHYLLQPHGAPTWGAIPKLPPPGRSPLAQRPLRRQ 2045

464 AQSGSILSVHSQPADTSCILQLPKDAHYLLQPHGAPTWGAIPKLPPPGRSPLAQRPLRRQ 998

785 AQSGSILSVHSQPADTSCILQLPKDAHYLLQPHGAPTWGAIPKLPPPGRSPLAQRPLRRQ 2005

784 AQSGSILSVHSQPADTSCILQLPKDAHYLLQPHGAPTWGAIPKLPPPGRSPLAQRPLRRQ 2022

783 AQSGSILSVHSQPADTSCILQLPKDAHYLLQPHGAPTWGAIPKLPPPGRSPLAQRPLRRQ 2029

965 AQSGSILSVHSQPADTSCILQLPKDAHYLLQPHGAPTWGAIPKLPPPGRSPLAQRPLRRQ 575

865 AQSGSILSVHSQPADTSCILQLPKDAHYLLQPHGAPTWGAIPKLPPPGRSPLAQRPLRRQ 483

113 AQSGSILSVHSQPADTSCILQLPKDAHYLLQPHGAPTWGAIPKLPPPGRSPLAQRPLRRQ 482

465 AAIRTDSLDVQGLGSREDLLSEVSGPSCPLTRSSSFWGGSSIQVQQRSGSQSKVSKHIRL 1267

463 AAIRTDSLDVQGLGSREDLLSEVSGPSCPLTRSSSFWGGSSIQVQQRSGSQSKVSKHIRL 315

966 AAIRTDSLDVQGLGSREDLLSEVSGPSCPLTRSSSFWGGSSIQVQQRSGSQSKVSKHIRL 2105

464 AAIRTDSLDVQGLGSREDLLSEVSGPSCPLTRSSSFWGGSSIQVQQRSGSQSKVSKHIRL 1058

785 AAIRTDSLDVQGLGSREDLLSEVSGPSCPLTRSSSFWGGSSIQVQQRSGSQSKVSKHIRL 2065

784 AAIRTDSLDVQGLGSREDLLSEVSGPSCPLTRSSSFWGGSSIQVQQRSGSQSKVSKHIRL 2082

783 AAIRTDSLDVQGLGSREDLLSEVSGPSCPLTRSSSFWGGSSIQVQQRSGSQSKVSKHIRL 2089

965 AAIRTDSLDVQGLGSREDLLSEVSGPSCPLTRSSSFWGGSSIQVQQRSGSQSKVSKHIRL 635

865 AAIRTDSLDVQGLGSREDLLSEVSGPSCPLTRSSSFWGGSSIQVQQRSGSQSKVSKHIRL 543

113 AAIRTDSLDVQGLGSREDLLSEPLAQAWNPAGPRTLKRPEAA------------------ 524

465 PAPCPGLEPSWAKDPQETRSSLELDTELSWISGDLLPSSQEEPLSPRDLKKCYSVEAQSC 1327

463 PAPCPGLEPSWAKDPQETRSSLELDTELSWISGDLLPSSQEEPLSPRDLKKCYSVEAQSC 375

966 PAPCPGLEPSWAKDPQETRSSLELDTELSWISGDLLPSSQEEPLSPRDLKKCYSVEAQSC 2165

464 PAPCPGLEPSWAKDPQETRSSLELDTELSWISGDLLPSSQEEPLSPRDLKKCYSVEAQSC 1118

785 PAPCPGLEPSWAKDPQETRSSLELDTELSWISGDLLPSSQEEPLSPRDLKKCYSVEAQSC 2125

784 PAPCPGLEPSWAKDPQETRSSLELDTELSWISGDLLPSSQEEPLSPRDLKKCYSVEAQSC 2142

783 PAPCPGLEPSWAKDPQETRSSLELDTELSWISGDLLPSSQEEPLSPRDLKKCYSVEAQSC 2149

965 PAPCPGLEPSWAKDPQETRSSLELDTELSWISGDLLPSSQEEPLSPRDLKKCYSVEAQSC 695

865 PAPCPGLEPSWAKDPQETRSSLELDTELSWISGDLLPSSQEEPLSPRDLKKCYSVEAQSC 603

465 RRRPGSWLDEQRRHSIAVSCLDSGSQPRLCPSPSSLGGQPLGGPGSRPKKKLSPPSISID 1387

463 RRRPGSWLDEQRRHSIAVSCLDSGSQPRLCPSPSSLGGQPLGGPGSRPKKKLSPPSISID 435

966 RRRPGSWLDEQRRHSIAVSCLDSGSQPRLCPSPSSLGGQPLGGPGSRPKKKLSPPSISID 2225

464 RRRPGSWLDEQRRHSIAVSCLDSGSQPRLCPSPSSLGGQPLGGPGSRPKKKLSPPSISID 1178

785 RRRPGSWLDEQRRHSIAVSCLDSGSQPRLCPSPSSLGGQPLGGPGSRPKKKLSPPSISID 2185

784 RRRPGSWLDEQRRHSIAVSCLDSGSQPRLCPSPSSLGGQPLGGPGSRPKKKLSPPSISID 2202

783 RRRPGSWLDEQRRHSIAVSCLDSGSQPRLCPSPSSLGGQPLGGPGSRPKKKLSPPSISID 2209

965 RRRPGSWLDEQRRHSIAVSCLDSGSQPRLCPSPSSLGGQPLGGPGSRPKKKLSPPSISID 755

865 RRRPGSWLDEQRRHSIAVSCLDSGSQPRLCPSPSSLGGQPLGGPGSRPKKKLSPPSISID 663

465 PPESQGPRPPCSPGVCLRRRAPASDSKDPSASSPLDSTAASPSPKKDALSLSGLSSDPTD 1447

463 PPESQGPRPPCSPGVCLRRRAPASDSKDPSASSPLDSTAASPSPKKDALSLSGLSSDPTD 495

966 PPESQGPRPPCSPGVCLRRRAPASDSKDPSASSPLDSTAASPSPKKDALSLSGLSSDPTD 2285

464 PPESQGPRPPCSPGVCLRRRAPASDSKDPSASSPLDSTAASPSPKKDALSLSGLSSDPTD 1238

785 PPESQGPRPPCSPGVCLRRRAPASDSKDPSASSPLDSTAASPSPKKDALSLSGLSSDPTD 2245

784 PPESQGPRPPCSPGVCLRRRAPASDSKDPSASSPLDSTAASPSPKKDALSLSGLSSDPTD 2262

783 PPESQGPRPPCSPGVCLRRRAPASDSKDPSASSPLDSTAASPSPKKDALSLSGLSSDPTD 2269

965 PPESQGPRPPCSPGVCLRRRAPASDSKDPSASSPLDSTAASPSPKKDALSLSGLSSDPTD 815

865 PPESQGPRPPCSPGVCLRRRAPASDSKDPSASSPLDSTAASPSPKKDALSLSGLSSDPTD 723

465 LDP 1450

463 LDP 498

966 LDP 2288

464 LDP 1241

785 LDP 2248

784 LDP 2265

783 LDP 2272

965 LDP 818

865 LDP 726
